# Pyrrolidine-based 3-deoxysphingosylphosphorylcholine analogs as possible candidates against neglected tropical diseases (NTDs): identification of hit compounds towards development of potential treatment of *Leishmania donovani*

**DOI:** 10.1080/14756366.2021.1969385

**Published:** 2021-08-23

**Authors:** Ahmed H. E. Hassan, Trong-Nhat Phan, Seolmin Yoon, Cheol Jung Lee, Hye Rim Jeon, Seung-Hwan Kim, Joo Hwan No, Yong Sup Lee

**Affiliations:** a Department of Medicinal Chemistry, Faculty of Pharmacy, Mansoura University, Mansoura, Egypt; b Medicinal Chemistry Laboratory, College of Pharmacy, Kyung Hee University, Seoul, Republic of Korea; c Leishmania Research Laboratory, Institut Pasteur Korea, Seongnam-si, Korea; d Department of Life and Nanopharmaceutical Sciences, Kyung Hee University, Seoul, Republic of Korea

**Keywords:** Antileishmanial agents, promastigotes, amastigotes, repurposing, molecular docking, inositol phosphoceramide synthase, IPCS, sphingomyelin

## Abstract

A rational-based process was adopted for repurposing pyrrolidine-based 3-deoxysphingosylphosphorylcholine analogs bearing variable acyl chains, different stereochemical configuration and/or positional relationships. Structural features were highly influential on activity. Amongst, enantiomer **1e** having 1,2-vicinal relationship for the -CH_2_O- and the *N-*acyl moieties, a saturated palmitoyl chain and an opposite stereochemical configuration to natural sphingolipids was the most potent hit compound against promastigotes showing IC_50_ value of 28.32 µM. The corresponding enantiomer **1a** was 2-fold less potent showing a eudismic ratio of 0.54 in promastigotes. Compounds **1a** and **1e** inhibited the growth of amastigotes more potently relative to promastigotes. Amongst, enantiomer **1a** as the more selective and safer. *In silico* docking study using a homology model of *Leishmania donovani* inositol phosphoceramide synthase (IPCS) provided plausible reasoning for the molecular factors underlying the found activity. Collectively, this study suggests compounds **1a** and **1e** as potential hit compounds for further development of new antileishmanial agents.

## 1. Introduction

Neglected tropical diseases (NTDs) are mainly endemic to countries of the poorest populations of the world and, hence, there was low interest in developing treatments to combat this group of diseases^1^^,^^2^. Mostly, NTDs are infectious parasitic diseases. Among them, leishmaniasis emerges as a major NTD. Nearly, a population of more than one billion living in around 90 *Leishmania*-endemic countries are at risk of infection. Leishmaniasis can be clinically manifested in different forms including visceral leishmaniasis (VL; AKA kala-azar; the most serious and fatal form) that affects internal organs, cutaneous leishmaniasis (CL; the most common form) that causes skin sores, or mucocutaneous leishmaniasis (MCL; the most disabling form). About 1.5–2 million new cases and 70,000 deaths because of leishmaniasis are estimated per annum^3^. In fact, several *Leishmania*-infected persons are asymptomatic carriers showing no to sub-clinical symptoms and, thus, constitute a disease-reservoir and an added challenge to disease control and eradication efforts^4^.

**Figure 1.**
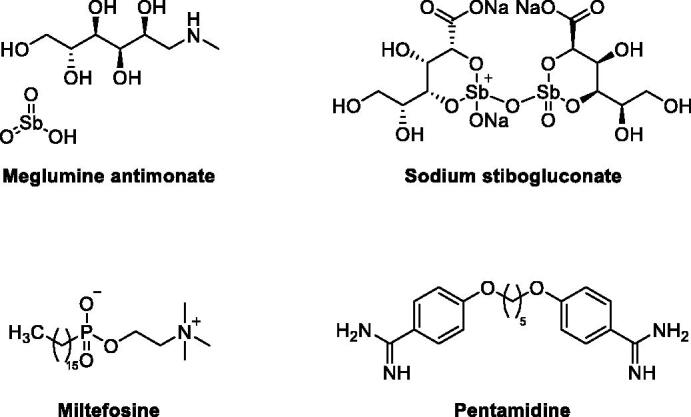
Example of currently used antileishmanial therapeutic agents.

Out of 53 known *Leishmania* species, 31 are mammals’ parasites including 20 human’s pathogenic species^5^. In lieu of the diversity of *Leishmania* parasites, it might be understandable that there is no available vaccine for protection, yet. Consequently, the development of an effective treatment remains the practically viable option for combating *Leishmania* infections. The current toolbox of antileishmanial therapeutic agents involves multiple classes of compounds (Figure 1) including antimony-containing compounds; such as meglumine antimonate and sodium stibogluconate, alkyl phospholipids; such as miltefosine, and amidine derivatives such as pentamidine. However, all of these treatments, especially against VL, suffer from tolerability and toxicity drawbacks such as cardiotoxicity, pancreatitis, hepatotoxicity, and nephrotoxicity of antimony-containing compounds; teratogenicity, nephrotoxicity and gastrointestinal effects of miltefosine; and diabetes mellitus; cardiotoxicity; nephrotoxicity or even death because of pentamidine. Furthermore, the emergence of drug resistance to the currently available treatments is a serious challenge threatening current efforts to cure the disease and curb infections. Accordingly, there is a real need for the development of new, less toxic, better tolerated, economic and effective treatments.

Repurposing, reprofiling, repositioning, or redirecting are closely correlated terms that have been coined to describe the attempt to deploy compound(s) for targeting a certain pathological condition(s) although originally they are/were used; developed to be used or failed the process of development to be used for another disease(s)^6–10^. Although such a strategy towards drug discovery and development was previously known, implemented, and indeed, resulted in successful discoveries, the current global threats because of pandemic COVID-19 increased the awareness and sparked more interest in this strategy. Advantageously, repurposing approaches require less development-timeline and resources in comparison with other drug discovery approaches. This might be evidently clear knowing that the outbreak of COVID-19, despite was in November 2019, it was not six months later until Institut Pasteur Korea, following repurposing strategy, discovered in May 2020 (published in August) that nafamostat and camostat are two potential candidates for the treatment of COVID-19; both are subject to ongoing clinical trials^11^^,^^12^. As of the premise of this approach, the employment of repurposing strategy was called on as an important tool to develop therapeutics for rare and neglected diseases (TRND) including parasitic protozoan diseases such as leishmaniasis^13–16^. Herein, we report our promising results for repurposing 3-deoxy-sphingosylphosphorylcholine (3-deoxy-SPC) analogs as antileishmanial agents.

## 2. Results and discussion

### 2.1. Repurposing rational of pyrrolidine-based 3-deoxy-SPC analogs

**Figure 2.**
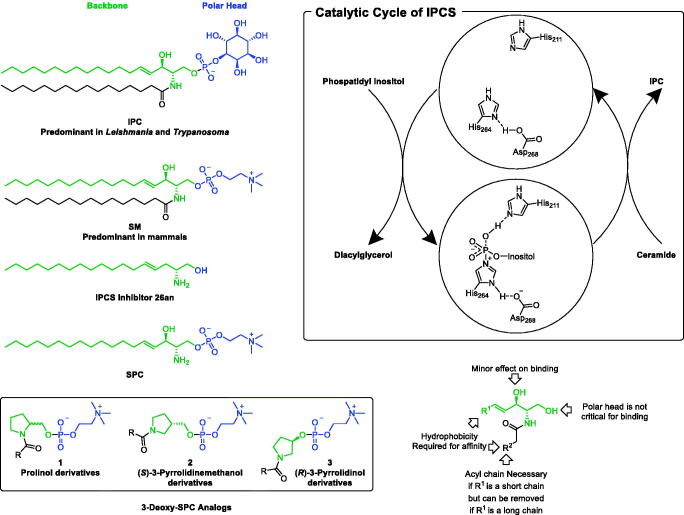
Rational of repurposing pyrrolidine-based 3-deoxy-SPC analogs for.

Sphingolipids (SLs), which constitute an important class of lipids, are crucial components of cellular membranes within mammalian cells; including human cells, and protozoan kinetoplastida including trypanosomatids such as *Trypanosoma* and *Leishmania*. However, inositol phosphoceramide (IPC; AKA inositol phosphorylceramide; Figure 2) is the major SL of trypanosomatids while it is not existent in mammalian cells which possess sphingomyelin (SM)-based SLs (Figure 2)^17–19^. Not only absent form mammalian’s SLs, but also IPC and the *Leishmania*’s machinery of SLs metabolism and synthesis play indispensable roles in *Leishmania*’s virulence and survival. *Leishmania*’s regulatory machine synthesises IPC via IPC synthase (IPCS) which possesses a phosphatase domain that exists also in other members of the lipid phosphate phosphatase (LPP) family of proteins^20^. This domain is necessary in IPCS to hydrolyse phosphatidylinositol in order to transfer the inositol phosphate moiety to ceramide to convert it into IPC (Figure 2). While the inhibition of IPCs was reported as a promising strategy towards the development of a new class of antileishmanial drugs^21^^,^^22^, scarce attempts are reported in the literature. Amongst, IPCS inhibitor **26an** (Figure 2), which possesses a single long alkyl chain and lacks the 3-hydroxy function, demonstrated significant cytotoxicity against *L. major* promastigotes^23^. In fact, a SAR study confirmed that the second acyl chain, the polar head, and the 3-hydroxy function are not critical for binding (Figure 2).

In lieu of these literature reports suggesting potential opportunities for the development of antileishmanial agents upon targeting the machinery involved in IPC synthesis, this work was initiated in search of a suitable hit compound. Recently, the synthesis of single chain-substituted pyrrolidine-based sphingolipid analogs has been described^24^. A retrospective view of these compounds suggests that these compounds might satisfy the postulated requirements to be considered as 3-deoxy-sphingosylphosphorylcholine analogs (3-deoxy-SPC analogs, Figure 2) of sphingosylphosphorylcholine (SPC; AKA sphingosine phosphocholine or lysosphingomyelin); a single chain des-acyl sphingomyelin that shares similar structural features to IPCS inhibitor **26an**, in combination with the presence of a phosphocholine polar head characteristic for sphingomyelins. Tentatively, 3-deoxy-SPC analogs of SPC might compete with IPCS substrates and, in addition, if underwent hydrolysis in the first step of the catalytic cycle would provide phosphocholine instead of phosphoinositol. Ultimately, they might block the synthesis of IPC. Accordingly, 3-deoxy-SPC analogs of SPC might be good candidates for the development of antileishmanial agents. Consequently, this work aimed to explore the possibility of repurposing these previously reported compounds for the treatment of leishmaniasis. In this regard, re-synthesis and investigation of the impact of 3-deoxy-SPC analogs on the viability of promastigotes of *L. donovani*; the most fatal species of *Leishmania*, was conducted. As of the chirality of the pyrrolidine-based 3-deoxy-SPC analogs, the current work considered evaluation of compounds retaining the 1,2-vicinal relationship for the -CH_2_O- and the *N-*acyl moieties and possessing a chiral configuration homologous to natural SLs or the opposite stereochemical configuration (D- and L-prolinol derivatives **1**, Figure 2). In addition, the impact of increasing the distance between the nitrogen and the oxygen to afford derivatives with 1,3-vicinal relationship for the -CH_2_O- and the *N-*acyl moieties was planned to be explored ((*S*)-3-pyrrolidinemethanol derivatives **2**, Figure 2) or directly bonding the oxygen to the pyrrolidine ring ((*R*)-3-pyrrolidinol derivatives **3**, Figure 2) were planned to be explored. Towards the assessment of the impact of variation of the size and shape of the acyl chains, various saturated and *cis* unsaturated acyl chains were incorporated included palmitoyl, palmitoleoyl, oleoyl, erucoyl, linoleoyl, and α-linolenoyl moieties. Needless to mention that introduction of a *cis* unsaturated double bond results in a kink in the chain configuration per each introduced double bond.

### 2.2. Chemistry (synthesis)

**Scheme 1.**
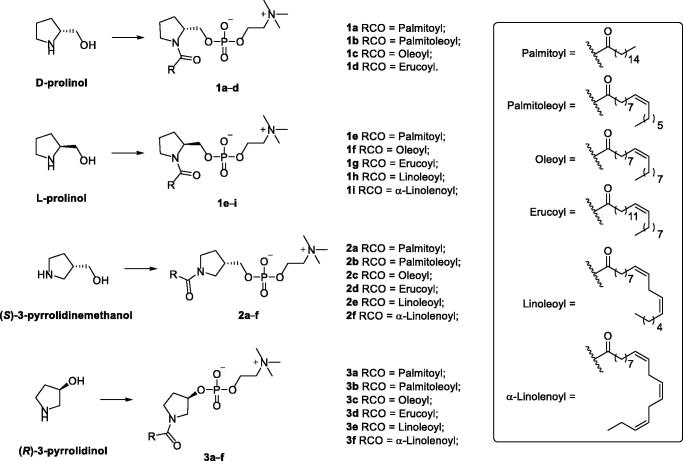
Reagents and conditions: (a) NEt_3_, DMF, rt; (b) 2-chloro-2-oxo-1,3,2-dioxaphospholane, NEt_3_, benzene, 0 °C to rt, (c) NMe_3_, CH_3_CN, 60 °C

The synthesis of pyrrolidine-based 3-deoxy-SPC analogs was performed in three consecutive steps as reported earlier^24^ starting from an already known chiral pool of pyrrolidine-alcohol derivatives and fatty acid chlorides. Derivatives **1a–d**, possessing the same stereochemical configuration of natural SPC and SLs, were synthesised from D-prolinol which was acylated with the appropriate acyl chloride, then esterified with 2-chloro-2-oxo-1,3,2-dioxaphospholane followed by reaction with trimethylamine to afford the desired pyrrolidine-based 3-deoxy-SPC analogs **1a–d** (Scheme 1). Similarly, the reaction of L-prolinol with appropriate acyl chloride following the same three consecutive steps afforded desired pyrrolidine-based 3-deoxy-SPC analogs **1e–i** whose stereochemical configuration is opposite to natural SPC and SLs (Scheme 1). Analogously, reacting (*S*)-3-pyrrolidinemethanol or (*R*)-3-pyrrolidinol as the pyrrolidine moiety yielded derivatives **2a–f** with 1,3-vicinal relationship for the -CH_2_O- and the *N-*acyl moieties or derivatives **3a–f** with directly bonded oxygen to the pyrrolidine nucleus, respectively (Scheme 1).

### 2.3. Evaluation of antileishmanial activity

#### 2.3.1. *In vitro* evaluation against *L. donovani* promastigotes at 50 and 25 µM single doses

Out of 20 known *Leishmania* species pathogenic to humans, *L. donovani*, which causes visceral leishmaniasis, is the most fatal^25^. Consequently, an *in vitro* model of *L. donovani* promastigotes^26^ was selected to evaluate the activity of the synthesised pyrrolidine-based 3-deoxysphingosylphosphorylcholine analogs **1e–i**, **2a–f**, **3a–f** at two concentration (50 and 25 µM) employing miltefosine and erufosine (ErPC; erucylphosphocholine) as reference standards using a resazurin-based assay^27^. The results are summarised in Table 1.

**Table 1.** *In vitro* evaluation results for inhibition of growth of *L*.* donovani* promastigotes.

Compound	Acyl chain type	Characteristic structural features of acyl chain	% Inhibition at 50 µM concentration^a^	% Inhibition at 25 µM concentration^a^
**1a**	Palmitoyl	Saturated	108	107
**1b**	Palmitoleoyl	C16:1 cisΔ^9^	109	108
**1c**	Oleoyl	C18:1 cisΔ^9^	51	10
**1d**	Erucoyl	C22:1 cisΔ^13^	108	107
**1e**	Palmitoyl	Saturated	107	107
**1f**	Oleoyl	C18:1 cisΔ^9^	108	65
**1g**	Erucoyl	C22:1 cisΔ^13^	109	110
**1h**	Linoleoyl	C18:2 cisΔ^9,12^	0	−18
**1i**	α-Linolenoyl	C18:3 cisΔ^9,12,15^	1	−16
**2a**	Palmitoyl	Saturated	108	107
**2b**	Palmitoleoyl	C16:1 cisΔ^9^	98	27
**2c**	Oleoyl	C18:1 cisΔ^9^	107	−19
**2d**	Erucoyl	C22:1 cisΔ^13^	109	107
**2e**	Linoleoyl	C18:2 cisΔ^9,12^	101	34
**2f**	α-Linolenoyl	C18:3 cisΔ^9,12,15^	9	0
**3a**	Palmitoyl	Saturated	109	108
**3b**	Palmitoleoyl	C16:1 cisΔ^9^	90	67
**3c**	Oleoyl	C18:1 cisΔ^9^	106	106
**3d**	Erucoyl	C22:1 cisΔ^13^	104	107
**3e**	Linoleoyl	C18:2 cisΔ^9,12^	69	0
**3f**	α-Linolenoyl	C18:3 cisΔ^9,12,15^	1	−14
**Miltefosine**	–	–	100	100
**Erufosine**	–	–	108	106

^a^ % Inhibition of growth of *L*.* donovani* promastigotes after incubation with the specified concentration for 3 days relative to control.

Investigation of the results unveiled an interesting activity pattern linked to the structural features of the acyl chains. Among pyrrolidine-based 3-deoxy-SPC analogs **1a–d** possessing chiral configuration homologous to natural SLs and 1,2-vicinal relationship for the -CH_2_O- and the *N-*acyl moieties, the saturated palmitoyl derivative (**1a**), the C16:1 cisΔ^9^ palmitoleoyl derivative (**1 b**) and the C22:1 cisΔ^13^ erucoyl derivative (**1d**), but not the C18:1 cisΔ^9^ oleoyl derivative (**1c**) produced ≥ 100 inhibition at both 50 and 25 µM concentrations (similar to reference drugs miltefosine and erufosine; Table 1). Despite that the C16:1 cisΔ^9^ palmitoleoyl derivative (**1 b**) and the C18:1 cisΔ^9^ Oleoyl derivative (**1c**) have the same double bond position, the much lower activity of the latter might be attributed to its increased chain length. The significant high activity of the C22:1 cisΔ^13^ erucoyl derivative (**1d**) although its longer chain lengths might arise from the different kink position as its unsaturated double bond exists at Δ^13^ instead of Δ^9^ position.

A more or less similar pattern was identified for pyrrolidine-based 3-deoxy-SPC analogs **1e–i** that possess an opposite chiral configuration to natural SLs but retain the 1,2-vicinal relationship for the -CH_2_O- and the *N-*acyl moieties as analogs **1a–d**. The saturated palmitoyl derivative (**1e**) and the unsaturated C22:1 cisΔ^13^ erucoyl derivative (**1 g**) were highly active at both 50 and 25 µM concentrations. Meanwhile, the activity of unsaturated C18:1 cisΔ^9^ oleoyl derivative (**1f**) decreased sharply at 25 µM relative to 50 µM concentration. Surprisingly, the polyunsaturated C18:2 cisΔ^9,12^ linoleoyl derivative (**1 h**) and C18:3 cisΔ^9,12,15^ α-linolenoyl derivative (**1i**) were completely inactive at both 50 and 25 µM concentrations. This outcome suggests intolerance of the combination of L-prolinol nucleus and rigidification of the acyl chain in a multiple-twisted configuration at these positions.

Activity trend of (*S*)-3-pyrrolidinemethanol derivatives **2a–f** having an extra carbon spacer, thus have 1,3-vicinal relationship for the -CH_2_O- and the *N-*acyl moieties showed partial similarity relative to analogs **1e–i**. Thus, the saturated palmitoyl derivative (**2a**) and the unsaturated C22:1 cisΔ^13^ erucoyl derivative (**2d**) were highly active at both 50 and 25 µM concentrations (similar to palmitoyl and erucoyl derivative among analogs **1e–i**). In addition, the activity of unsaturated C18:1 cisΔ^9^ oleoyl derivative (**2c**) decreased sharply at 25 µM relative to 50 µM concentration (analogous to oleoyl derivative (**1f**)) Meanwhile; the C18:3 cisΔ^9,12,15^ α-linolenoyl derivative (**2f**) was completely inactive at both concentrations. In contrast to the high activity of C16:1 cisΔ^9^ palmitoleoyl derivative (**1 b**), the activity of palmitoleoyl derivative (**2 b**) declined sharply at 25 µM concentration (Table 1). Furthermore, the C18:2 cisΔ^9,12^ linoleoyl derivative (**2e**), despite its low activity at 25 µM concentration, showed high activity at 50 µM concentration (unlike the complete inactivity of C18:2 cisΔ^9,12^ linoleoyl derivative (**1 h**)). Exploring (*R*)-3-pyrrolidinol nucleus, 3-deoxy-SPC analogs **3a–f** having directly bonded oxygen to the pyrrolidine nucleus revealed that the palmitoyl derivative (**3a**) and the unsaturated C22:1 cisΔ^13^ erucoyl derivative (**3d**) were highly active at both 50 and 25 µM concentrations. Different from the rapid decline in activity of oleoyl derivatives **1c**, **1f** and **2c**, the C18:1 cisΔ^9^ oleoyl derivative (**3c**) maintained high activity at both of 50 and 25 µM concentrations. Like palmitoleoyl derivative (**2 b**) but different from palmitoleoyl derivative (**1 b**), the activity of palmitoleoyl derivative (**3 b**) was high at 50 µM concentration, however, it rapidly declined at 25 µM concentration. In addition to the low linoleoyl derivative (**3e**) at 50 µM concentration, it showed a further sharp decline at 25 µM concentration (similar behaviour to linoleoyl derivative **2e**). Consistent with the inactivity of α-linolenoyl derivatives **1i** and **2f**, the α-linolenoyl derivative (**3f**) was completely inactive. This outcome bolsters the suggested intolerance to the rigidification of the acyl chain via the introduction of multiple unsaturation at these sites.

#### 2.3.2. *In vitro* potency evaluation for the most active compounds against *L. donovani* promastigotes

As most of the tested compounds showed inhibitory activity at 25 and 50 µM similar to Erufosine and Miltefosine and in order to assess the quality of identified hit compounds, a more rigorous dose-dependent activity evaluation was conducted. The lower doses used for the generation of the dose-response curve would differentiate between potent and less potent compounds and, thus, would allow assessing the potency of the compounds that maintained ≥ 100 inhibition at both 50 and 25 µM concentrations in the previous double concentrations assay. For comparison, erufosine and miltefosine were used as reference standards. The results are summarised in Table 2.

**Table 2.** *In vitro* evaluation results of IC_50_ values for growth of *L*.* donovani* promastigotes

Compound	Acyl Chain	IC_50_ ± SD (µM)^a^	Compound	Acyl chain	IC_50_ ± SD (µM)^a^
**1a**	Palmitoyl	52.59 ± 1.44	**2a**	Palmitoyl	54.61 ± 2.02
**1b**	Palmitoleoyl	66.13 ± 3.61	**2d**	Erucoyl	>100
**1d**	Erucoyl	46.65 ± 0.45	**3a**	Palmitoyl	>100
**1e**	Palmitoyl	28.32 ± 1.38	**3c**	Oleoyl	>100
**1g**	Erucoyl	68.47 ± 0.04	**3d**	Erucoyl	>100
**Erufosine**	–	9.75 ± 2.41	**Miltefosine**	–	4.94 ± 0.47

^a^Concentration was calculated from the dose response curves that inhibit the growth of *L*.* donovani* promastigotes by 50% after incubation for 3 days, relative to control.

Consistent with and not far from literature reported IC_50_ values for erufosine and miltefosine against *L. donovani* promastigotes, determined IC_50_ values for the standard erufosine and miltefosine were 9.75 and 4.94 µM, respectively (reported 13.3 and 3.1 µM, respectively)^28^. Interestingly, the results of potency evaluation showed that both the structural features of the acyl chains and the stereochemical configuration of the pyrrolidine ring as potency influential factors. In general, the pyrrolidine-based 3-deoxy-SPC analogs possessing saturated palmitoyl moiety as the acyl counterpart showed appreciable potency for all of the investigated pyrrolidine-based cores, except for pyrrolidine-based core **3**. Notably, derivative **1e** whose stereochemical configuration is opposite to natural SPC and SLs was the most potent hit compound. It showed IC_50_ value of 28.32 µM, which is nearly one-third the potency of erufosine and one-sixth the potency of miltefosine, respectively. This potency is two-fold the potency of derivative **1a** which preserved a similar stereochemical configuration of natural SPC and SLs. Derivative **2a** combining the saturated palmitoyl moiety with 1,3-vicinal relationship for the -CH_2_O- and the *N-*acyl moieties showed potency not far from the potency of derivative **1a**. Meanwhile, derivative **3a** combining the saturated palmitoyl moiety with direct bonding of the oxygen to the pyrrolidine ring showed weak potency. On the other side, a combination of the unsaturated C22:1 cisΔ^13^ erucoyl moiety with the pyrrolidine core possessing 1,3-vicinal relationship for the -CH_2_O- and the *N-*acyl moieties (derivative **2d**) or direct bonding of the oxygen to the pyrrolidine ring (derivative **3d**) resulted in a poor potency. Nevertheless, erucoyl derivatives **1d** and **1 g** possessing pyrrolidine core characterised by 1,2-vicinal relationship for the -CH_2_O- and the *N-*acyl moieties demonstrated relatively comparable potencies to palmitoyl derivative **1a** (IC_50_ values of 46.65, 68.47 and 52.59 µM for derivatives **1d**, **1g,** and **1a**, respectively). Relative to derivative **1a** combining the saturated palmitoyl chain with a pyrrolidine core characterised by 1,2-vicinal relationship for the -CH_2_O- and the *N-*acyl moieties, the potency of the analogous derivative **1b** possessing the monounsaturated the palmitoleoyl moiety showed a slight reduction in potency. Finally, the potency of derivative **3c** combining the monounsaturated C18:1 cisΔ^9^ oleoyl moiety with direct bonding of the oxygen to the pyrrolidine ring demonstrated a weak potency. Collectively, these results indicate that compounds derived from pyrrolidine core 1, characterised by 1,2-vicinal relationship for the -CH_2_O- and the *N-*acyl moieties, were the most promising derivatives, while those derived from pyrrolidine core 3, characterised by direct bonding of oxygen to the pyrrolidine ring, were impotent compounds. In addition, the results presented palmitoyl derivative **1e** whose stereochemical configuration is opposite to the natural SPC and SLs as the most potent amongst evaluated compounds. In addition, compound **1e** establishes a eutomer/distomer relationship with palmitoyl derivative **1a** showing a eudismic ratio of 0.54.

#### 2.3.3. *In vitro* evaluation against amastigotes of *L. donovani*, selectivity and safety

As the extracellular promastigotes form represents the insect stage of the parasite, assessment of activity is needed to confirm activity against the intracellular amastigotes form, which is the human host form (mammalian host form). In addition, a preliminary assessment of selectivity and safety might be desirable to evaluate the quality of hit compounds. Accordingly, compound **1e** and its enantiomer; **1a**, in parallel with standard drugs miltefosine and erufosine were submitted for evaluation in intracellular amastigotes model using infected THP-1 cells; human monocytic cell line derived from an acute monocytic leukaemia. In addition, cytotoxicity of tested compounds and standards were evaluated against THP-1 cells and normal human embryonic kidney cells; HEK293T to evaluate selectivity and safety. The results are summarised in Table 3.

**Table 3.** Efficacy of tested compounds in cells infected with intracellular *L. donovani,* and cytotoxicity against THP-1 and HEK293T cells.

Compounds	Intracellular *L. donovani* IC_50_ ± SD^a^ (μM)	Extracellular *L. donovani* Promastigotes IC_50_ ± SD^a^ (μM)	THP-1 cell CC_50_± SD^a^ (μM)	HEK293T CC_50_ ± SD^a^ (μM)	Selectivity index^b^	Safety index^c^
**1a**	11.32 ± 1.81	52.59 ± 1.44	25.28 ± 3.56	30.64 ± 2.31	2.2	2.7
**1e**	14.31 ± 2.03	28.32 ± 1.38	15.78 ± 3.23	45.10 ± 5.01	1.1	3.2
**Erufosine**	3.61 ± 1.05	9.75 ± 2.41	3.94 ± 1.02	40.80 ± 3.36	1.1	11.3
**Miltefosine**	2.55 ± 0.63	4.94 ± 0.47	40.84 ± 3.32	>200	16.0	>78.4

^a^Shown are mean EC_50_ ± SD (standard deviations) of data from duplicate measurements.

^b^Selectivity index was calculated by dividing determined CC_50_ against human monocytic cell line derived from an acute monocytic leukaemia; THP-1 by determined IC_50_ for inhibition of Intracellular *L. donovani*.

^c^Safety index was calculated by dividing determined CC_50_ against normal human embryonic kidney cells; HEK293T by determined IC_50_ of Intracellular *L. donovani*.

The obtained results showed that both of tested compounds as well as the standard drugs were more potent inhibitors of the growth of amastigotes form of *L. donovani* relative to the promastigotes form. Interestingly, a dramatic decrease in IC_50_ against amastigotes form was more prominent in the case of enantiomer **1a** (4.64-folds for enantiomer **1a** and 1.98-folds for enantiomer **1e**). This triggered reversal of potency order in amastigotes rendering enantiomer **1a** more potent inhibitors. A presence of an additional functioning mechanism of action more pronounced in amastigotes might be behind this change in activity trend. The measured cytotoxicity against THP-1 cells derived from acute monocytic leukaemia that served as host cells for amastigotes showed 2.2 selectivity index for compound **1a** that is better than the selectivity of erufosine but lower than the selectivity of miltefosine. Despite, the low selectivity index of compound **1e**, its index is comparable with the standard drug erufosine. Although cytotoxicities of both compound **1a** and **1e** against normal human embryonic kidney cells; HEK293T were comparable to the standard erufosine, their relatively lower potencies rendered their safety index lower than the standard drugs. However, these collective results confirm enantiomers **1a** and **1e** eliciting antileishmanial activity against both *L. donovani* amastigotes (mammalian host form) and promastigotes (insect form). Amongst them, enantiomer 1a is a more selective and potent inhibitor of the growth of *L. donovani* amastigotes.

### 2.4. Molecular modeling study

Towards performing *in silico* study, a 3D coordinates file of IPCS of *L. donovani*, which served as the rational for repurposing, to predict the possible binding mode with it. The most active hit **1e** whose stereochemical configuration is opposite to natural SPC and SLs as well as its corresponding enantiomer; compound **1a** was employed to investigate their possible interactions with IPCS. The results are summarised in the following sections.

#### 2.4.1. Homology modelling

As no resolved crystal structure is available yet for *L. donovani* IPCS, building a 3D homology model (uniport sequence ID: E9BT22) was conducted. It is a fact that IPCS shares with other members of lipid phosphate phosphatase (LPP) family of proteins a homologous phosphatase domain characterised with conserved residues. Especially, a conserved common catalytic triad (His211/His264/Asp268 of *L. donovani* IPCS homologous, for example, to His163/His207/Asp211 of Phosphatase phosphatidylglycerophosphate phosphatase B (PgpB); PDB IDs: 5jwy and 4px7, exists in TM3 (His211 or His163) and TM6 (His264/Asp268 or His207/Asp211) nearby the periplasm^29^^,^^30^. In addition to the six transmembrane helices of PgpB, the 3D structure involves four extracellular helices (EH1–4) connecting TM3 and TM4 as well as a cytoplasmic helix (CH1) after TM6.

**Figure 3.**
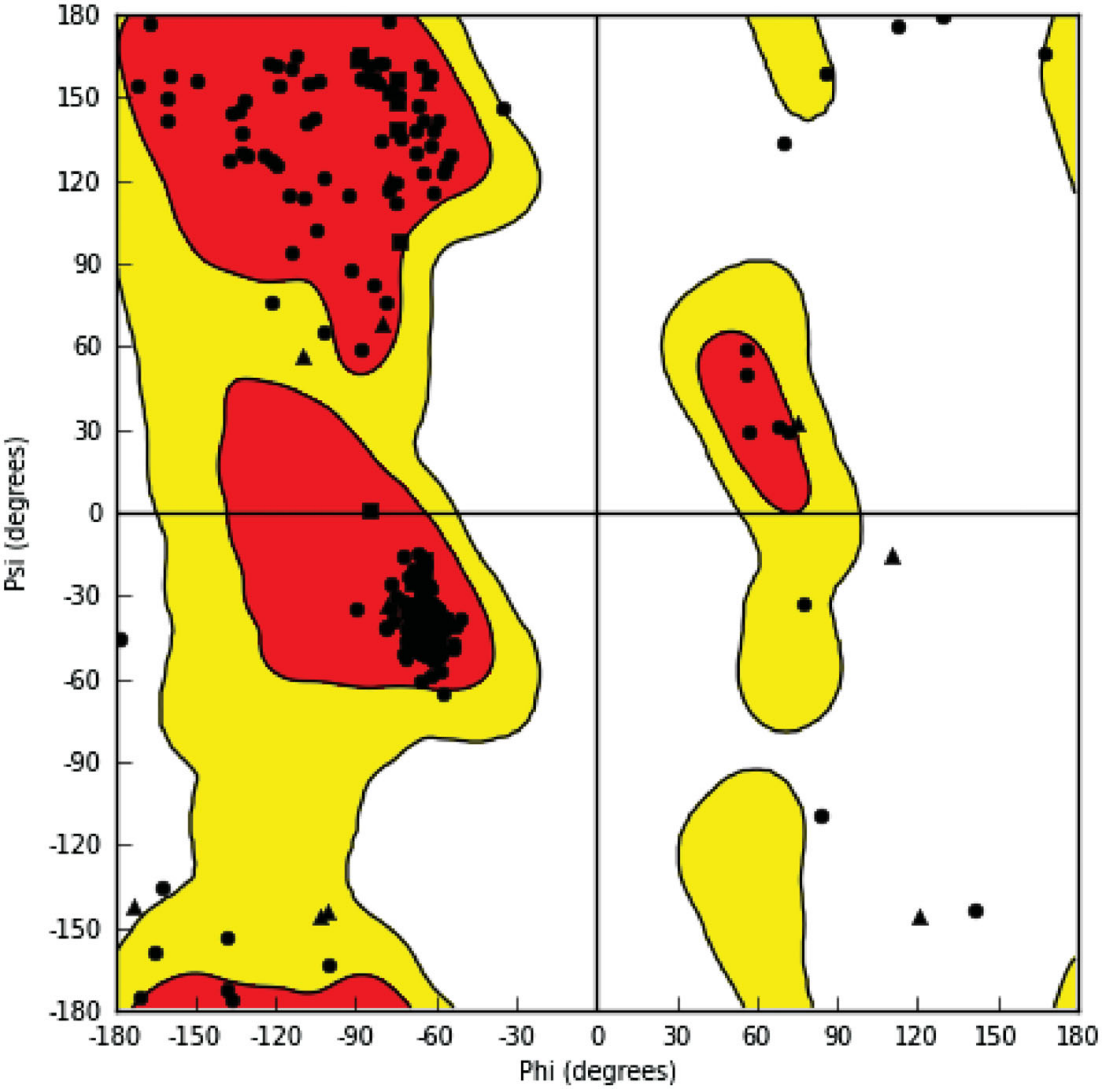
Ramachandran plot of the generated homology model of *L. donovani* IPCS.

In order to build the homology model, template search and sequence alignment were done employing homology detection and structure prediction using hidden Markov models (HMM) comparison using HHpred server^31^. Consistent with the known relation between IPCS and lipid phosphate phosphatase (LPP) family of proteins, the top hit retrieved by HHpred server was, phosphatase phosphatidylglycerophosphate phosphatase B (PgpB; PDB ID: 5jwy), with a calculated high probability (97.56%) and a low *E*-value (0.032). Accordingly, it was used as a template to build 3D homology model by MODELLER software (version 9.25). As shown in Figure 3, Ramachandran plot of φ/ψ of amino acid residues of the generated homology model indicated that most of them are located in the favoured regions. This indicates an acceptable quality for the generated model and thus can be employed in further studies.

#### 2.4.2. *In silico* docking study

**Figure 4.**
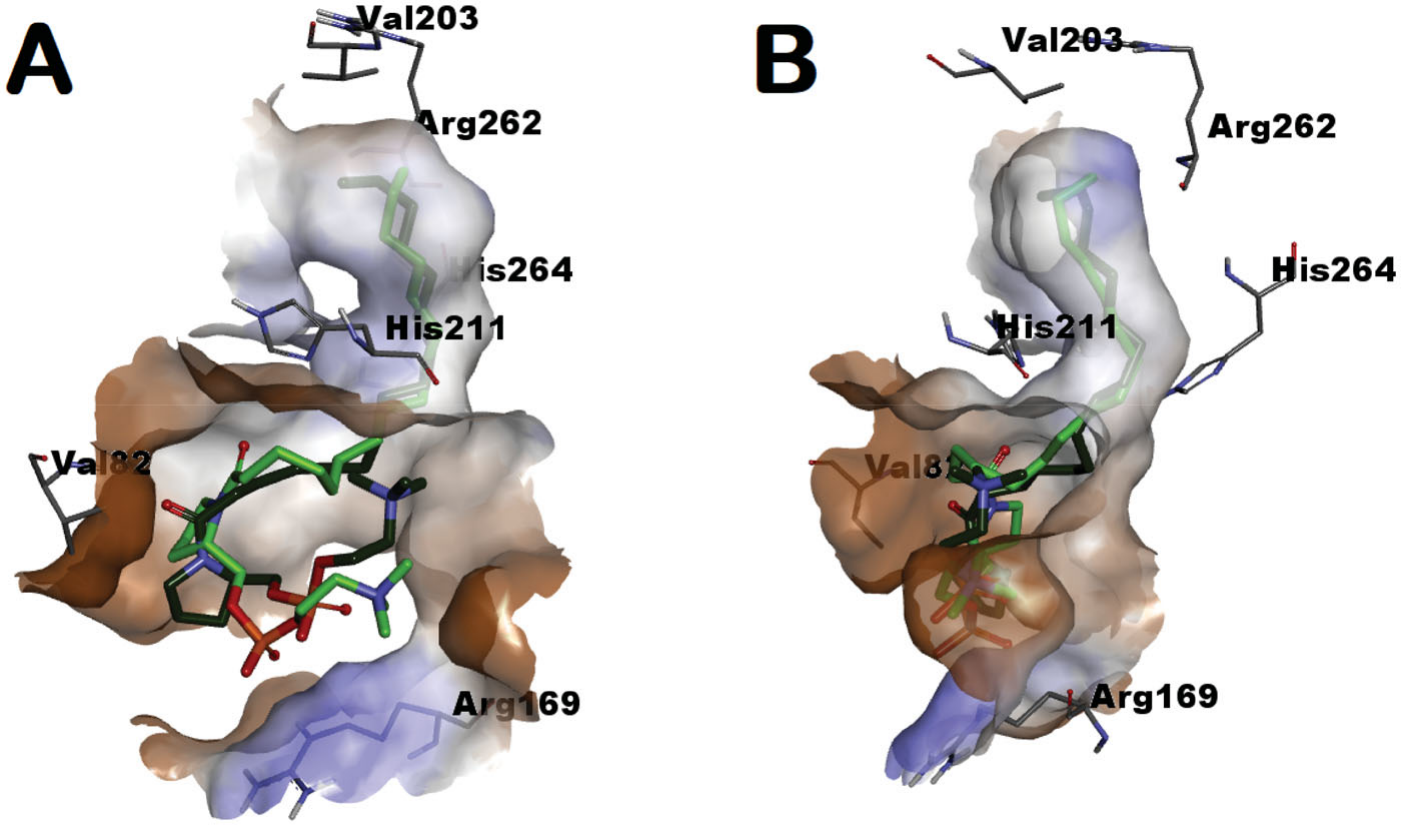
Enantiomers **1a** (dark green-colored carbon atoms) and **1e** (light green-colored carbon atoms) docked into the tunnel-shaped binding site of the generated homology model of *L. donovani* IPCS.

To get insights into the possible binding mode and interactions between the repurposed compounds and *L. donovani* IPCS, a docking study was conducted using the generated homology model after refinement with the most active hit compound **1e** whose stereochemical configuration is opposite to natural SPC and SLs as well as its corresponding enantiomer; compound **1a**. The proposed binding site was defined to span the catalytic triad residues His211/Asp268/His264 and the nearby residue Arg262. As shown in Figure 4, the binding site, in part, has a narrow tunnel connected to a relatively wide pore. Considering the best-obtained pose, both of the more active compounds **1e** and its less active enantiomer **1a** docked situating their saturated acyl chains into the narrow tunnel. This might explain the sensitivity of activity to chain kinks introduction in some unsaturated analogs as it might interfere with the fitting within this tunnel. As shown in Figure 4(B), the neck of the tunnel is formed by the space between the two catalytic residues His211 and His264. Accordingly, the calculated poses for the predicted binding mode of compounds **1a** and **1a** anticipate their ability to occupy this space between the catalytic residue and, thus, inhibit IPCS upon binding. In addition to the better docking score for compounds **1e** relative to **1a** (−8.45 relative to −8.07, respectively), compound **1e** showed also better energy for the docked conformer (−77.8344 and −65.3866 for docked conformers of compounds **1e** and **1a**, respectively). As the energy of a conformer reflects on its Boltzmann distribution and existence probability amongst the population of possible conformers, this might be translated into higher availability of the docked conformer of compound **1e** relative to the docked conformer of compound **1a**. Accordingly, both compounds **1e** and **1a** might be anticipated as inhibitors of *L. donovani* IPCS blocking its functions. Meanwhile, their found relation as eutomer/distomer might be understandable in lieu of these calculations.

## 3. Conclusion

As of our interest in developing therapeutics for rare and neglected infectious diseases, we followed a rational-based repurposing approach in an attempt to identify new hit compounds against the most fatal species of *Leishmania* parasites; the causative of VL (kala-azar). Based on the suggested rational, pyrrolidine-based 3-deoxy-SPC analogs might elicit potential antileishmanial activity. Accordingly, two steps *in vitro* evaluation process of pyrrolidine-based 3-deoxy-SPC analogs bearing variable acyl chains, different stereochemical configuration and/or positional relationships were performed. In the first step, compounds were evaluated at two different single doses. Molecules that maintained efficiency inhibiting the growth of *L. donovani* promastigotes at both concentrations were further evaluated for potency. The results showed that the most effective and potent derivatives are those based on pyrrolidine core 1, characterised by 1,2-vicinal relationship for the -CH_2_O- and the *N-*acyl moieties. Meanwhile, pyrrolidine core 3, characterised by direct bonding of oxygen to the pyrrolidine ring, did not provide promising compounds. In addition, the more rigid polyunsaturated acyl chains the poorest growth inhibition results were observed. Among investigated compounds, molecule **1e** bearing the saturated palmitoyl chain was identified as the most potent hit compound against promastigotes showing IC_50_ value of 28.32 µM. In addition, the results revealed a eutomer/distomer relationship between the two enantiomers **1e** and **1a** showing a eudismic ratio of 0.54 in promastigotes. However, enantiomer **1a** was a more potent inhibitor against amastigotes suggesting the presence of an additional operating mechanism in amastigotes but less pronounced in promastigotes. Evaluation of cytotoxicities suggested enantiomers **1a** and **1e** to elicit some degree of selectivity and safety. *In silico* docking study using a homology model for *L. donovani* IPCS presented a computational insight into the molecular factors that might mediate the observed variation in the bioactivity upon modification of the structure. Collectively, the current work presents compounds **1a** and **1e** as potential hits for further study and development towards accessing new antileishmanial agents.

## 4. Experimental

### 4.1. Chemistry

Compounds were synthesised as reported earlier and were in agreement with previously reported spectroscopic data^24^. Target compounds were purified using flash column chromatography (Stationary Phase: Silica gel; Mobile Phase: Chloroform/Methanol/Dist. Water = 6:25:4). The purity of all biologically evaluated compounds was assessed using HPLC Agilent 1100 series using SHISEIDO CAPCELL PAK C18 column (250   ×  4.6 mm, 5 μm) as a stationary phase employing an isocratic mobile phase composed of Methanol: Acetonitrile: Distilled water (85:15: 5 = v:v). HPLC purity testing showed purity ≥ 95% for all biologically evaluated compounds.

### 4.2. *In vitro* evaluation against *L. donovani* promastigotes

#### 4.2.1. Cell culture of parasite

*L. donovani* MHOM/SD/62/1S-CL2D parasites were cultured as promastigotes at 28 °C in M199 medium (Sigma-Aldrich, St. Louis, MO, USA) with 40 mM HEPES, 0.1 mM adenine, 0.0001% biotin, and 4.62 mM NaHCO_3_ supplemented with 10% foetal bovine serum (FBS, Gibco, Carlsbad, CA, USA), 100 µ/mL penicillin (Gibco), and 100 µg/mL streptomycin (Gibco). Parasites were sub-cultured every 3 or 4 days and maintained for 10 passages.

#### 4.2.2. Assay of parasite growth inhibition

The values of growth inhibition of *L. donovani* promastigotes were determined based on the metabolism of resazurin to resorufin by aerobic respiration of metabolically active cells using 384-well plates that were seeded with *L. donovani* promastigotes (5 × 10^4^ cells per well) and incubated with tested compounds for 3 days followed by addition of Resazurin sodium salt (200 μM; R7017; Sigma-Aldrich, St. Louis, MO, USA) and further incubation for 5 h then the cells were fixed (4% paraformaldehyde). The plates were analysed using a Victor3TM plate reader (PerkinElmer, Inc., Waltham, MA, USA) at 590 nm (emission) and 530 nm (excitation). Miltefosine and erufosine were used as the reference standards. All measured and calculated values are the averages of duplicates. The dose-response curves were generated by GraphPad Prism 6 software using a sigmoidal dose-response equation with a variable hill slope option.

### 4.3. *In vitro* evaluation against intracellular *L. donovani* amastigotes

PMA-treated THP-1 human monocytic cells were seeded at 0.8 × 10^4^ cells per well in a 384-well culture plate (Greiner Bio-One, Kremsmünster, Austria) in RPMI-1640 complete medium supplemented with 10% FBS. After 48 h of incubation at 37 °C in the presence of 5% CO2, infected THP-1 cells were treated with miltefosine (at 80 µM, positive control), and tested compounds (at 200 µM). The negative control consisted of THP-1 infected with the parasite with only 0.5% DMSO. After 72 h, the cells that were infected and treated with the drug were washed with serum-free RPMI-1640 medium. The cells and parasites were stained using 5 µM DAPI and 4% PFA. The images were acquired based on reading using an Operetta^®^ automated microscope (PerkinElmer, Inc., Waltham, MA 02451, USA). They were further analysed using Columbus^TM^ (PerkinElmer, Inc. Waltham, MA, USA) software to quantify parasite numbers, and host cell numbers. In brief, large-sized nucleus of host cells was first detected using DAPI signal and the host cell boundary masking was performed using the low-intensity signals from cytosols. Then the small-sized nucleus signal by DAPI was used to identify parasites within the area of the masked host cell. The DRC results were further assessed in a dose-dilution manner (two-fold serial dilution for 10 points starting from 200 µM).

### 4.4. Cytotoxicity evaluation against normal non-tumorigenic human cell

Cytotoxicity against HEK293T cell growth was assayed by measuring the conversion of resazurin to resorufin. The assays were performed in 384-well plates that were seeded with HEK293T cells (1000 cells per well). After seeding, the cells were exposed to the compounds for 3 days. Resazurin sodium salt (200 µM; R7017; Sigma-Aldrich, St. Louis, MO, USA) was then added, and the samples were incubated for 5 h. After incubation, the parasites were fixed using 4% paraformaldehyde, and the plates were analysed using a Victor3TM plate reader (PerkinElmer, Inc., Waltham, MA, USA) at 590 nm (emission) and 530 nm (excitation). Miltefosine and saponin were used as the reference drugs for the HEK293T growth inhibition.

### 4.5. *In silico* study

The amino acids sequence of *L. donovani* IPCS (UniProtKB - E9BT22, inositol phosphorylceramide synthase; gene: LDBPK_355030; https://www.uniprot.org/uniprot/E9BT22) was downloaded prom UniProt database of protein sequence and functional information. Template search and alignment was conducted using MPI Bioinformatics Toolkit (https://toolkit.tuebingen.mpg.de) using HHpred method. The 3D homology model was built using MODELLER software (version 9.25; https://salilab.org/modeller), refined and checked. The generated homology model was used for docking compounds **1a** and **1e**. The results were visualised by DS Visualiser.

## Supplementary Material

Supplemental Material
