# Supplementary material for: Pyrrolidine-based 3-deoxysphingosylphosphorylcholine analogs as possible candidates against neglected tropical diseases (NTDs): identification of hit compounds towards development of potential treatment of Leishmania donovani
Source: J Enzyme Inhib Med Chem. 2021 Aug 23;36(1):1922–30. doi: 10.1080/14756366.2021.1969385 (PMC8386730; doi:10.1080/14756366.2021.1969385)
Supplement: Supplemental Material [file IENZ_A_1969385_SM1637.pdf]

Supporting Information

Pyrrolidine-based 3-deoxysphingosylphosphorylcholine analogs as possible candidates against neglected tropical diseases (NTDs): Identification of hit compounds towards development of potential treatment of *Leishmania donovani*

Ahmed H.E. Hassan[a,b,1], Trong-Nhat Phan[c,1], Seolmin Yoon[d], Cheol Jung Lee[d], Hye Rim Jeon[d], Seung-Hwan Kim[d], Joo Hwan No[c*], Yong Sup Lee[b,d*]

[a] *Department of Medicinal Chemistry, Faculty of Pharmacy, Mansoura University, Mansoura 35516, Egypt.*

[b] *Medicinal Chemistry Laboratory, College of Pharmacy, Kyung Hee University, Seoul 02447, Republic of Korea.*

[c] *Leishmania Research Laboratory, Institut Pasteur Korea, Seongnam-si, Gyeonggi-do 13488, Korea.*

[d] *Department of Life and Nanopharmaceutical Sciences, Kyung Hee University, Seoul 02447, Republic of Korea.*

[1] Both of Ahmed H.E. Hassan and Trong-Nhat Phan have contributed equally. Accordingly, they should be recognized as co-first authors.

[*] **Corresponding authors:** Yong Sup Lee (e-mail: kyslee@khu.ac.kr) and Joo Hwan No (e-mail: joohwan.no@ip-korea.org).

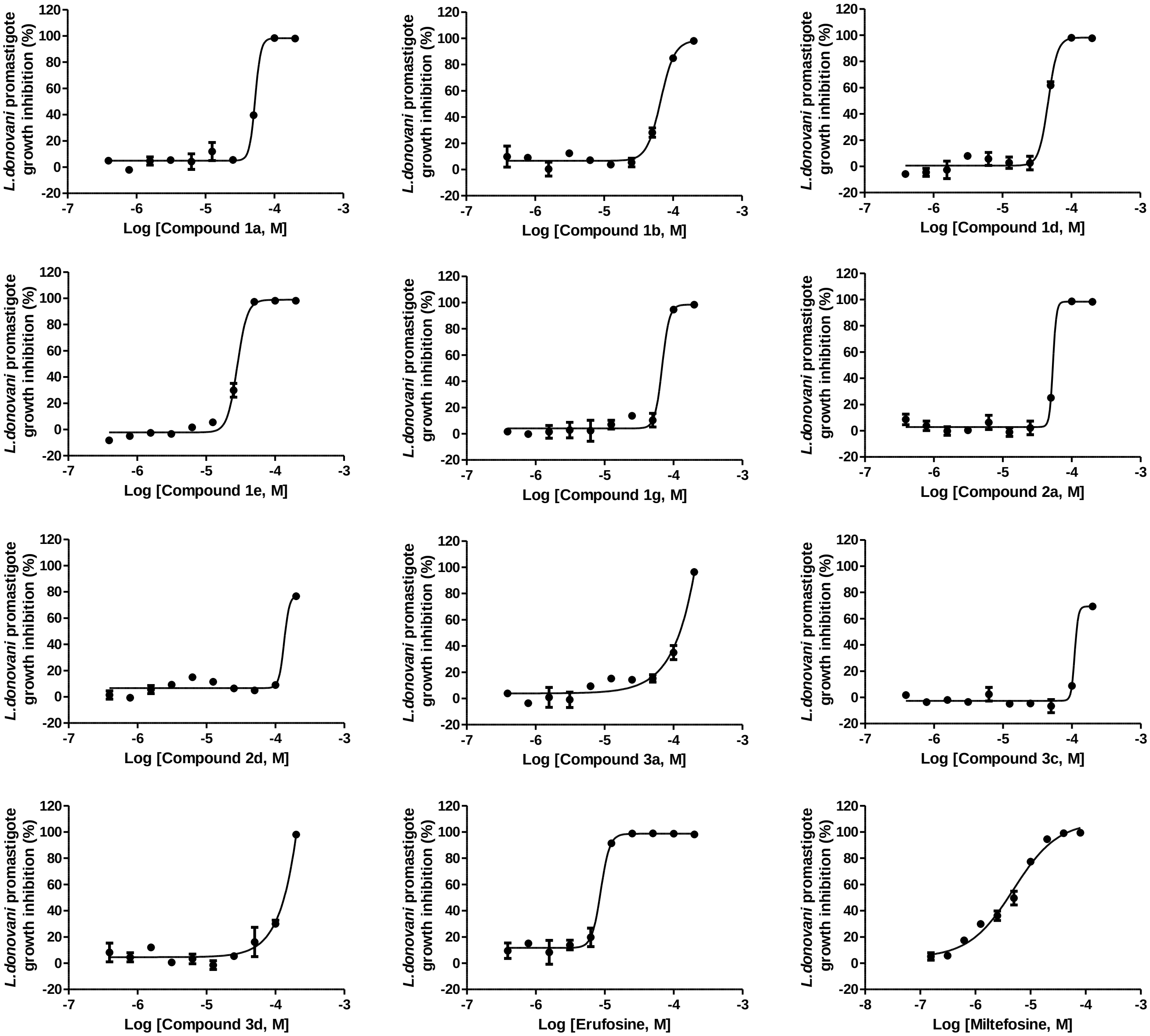

Dose-response curves of the standard erufosine, miltefosine and the hit compounds against *L. donovani* promastigote growth. The results are expressed as mean standard deviation values of duplicate experiments.

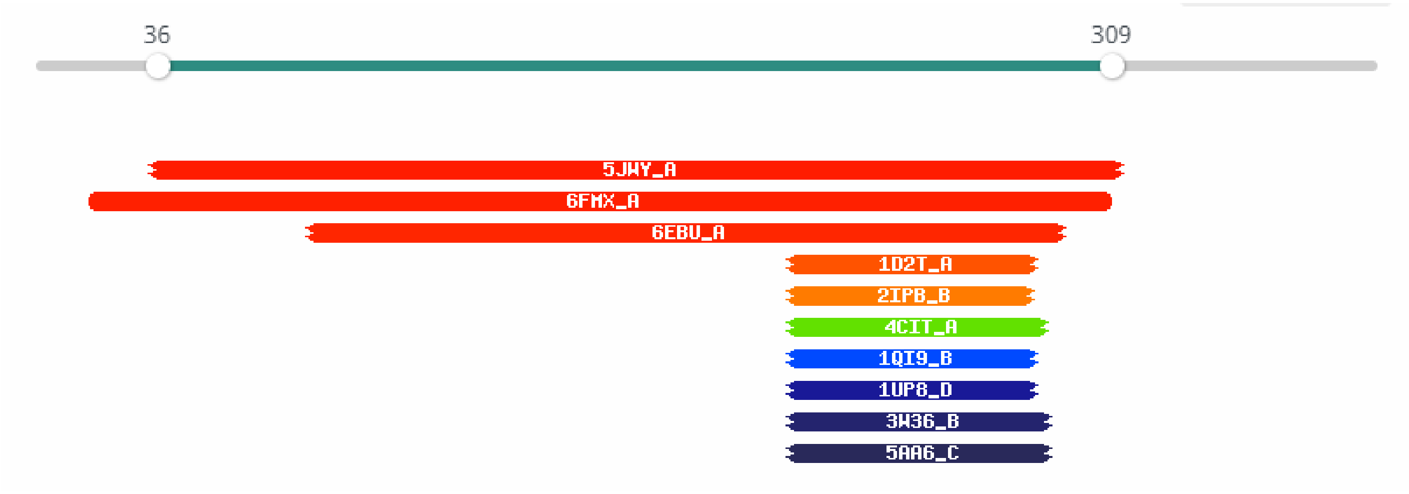

Templates retrieved for Sequence of IPCS of *Leishmania donovani* (Uniport ID: E9BT22)

Sequence alignment of IPCS of *Leishmania donovani* (Uniport ID: E9BT22) to template (PDB ID: 5JWY chain A)

5JWY_A Phosphatidylglycerophosphatase B (E.C.3.1.3.27, 3.1.3.81, 3.1.3.4; transmembrane helices, protein-lipid complex, enzyme; HET: MSE, 46E; 3.2A {Escherichia coli}; Related PDB entries: 4PX7_A

Probability: 97.56%, E-value: 0.032, Score: 52.75, Aligned cols: 215, Identities: 12%, Similarity: -0.096, Template Neff: 9.9

```
Q ss_pred         HHHHHHHHHHHHHHHHHHHHHHHHcCCCCccCCCCCchHHHhcCCCchhHHHHHHHHHHHHHHHHHHHHHHHHHHhccccCCC
Q 1            36 RFILLLLLTVMFLGVAILVANARMPDPKKVRPLPDLLLEWIPKVTLVENGSNVIIFLLNATTVVVGFKVFLLERHMNGLP   115 (385)
Q Consensus    36 r~~~~lli~~~i~~~a~~~~~~~r~Pd~~~~~PLpDl~fd~lP~i~~~~~i~d~~i~~l~~~~v~~~f~l~ll~r~r~~~~   115 (385)
                  +....+.+..++..+.....-..........+-.|-....+....... +....+.+..++ ..+..+.+
T Consensus     4 ~~~~~~~~~~~~~~~~~~~~~~~~~~~~~~~~~~~~~~~~~~~~~~~~~----~~~~~~~~~~~~----~~~~~~~~----   71 (259)
T 5JWY_A       4 IARRTAVGAALLLVMPVAVWISGWRWQPGEQSWLLKAAFWVTETVTQP----WGVITHLILFGW----FLWCLRFR----   71 (259)
T ss_dssp        HHHHHHHHHHHHHHHHHHHHHHHHHTCCCCSCCCHHHHHHHHHHHHHTTSTT----THHHHHHHHHHH----HHHHTGGG----
T ss_pred        HHHHHHHHHHHHHHHHHHHHHHHcCCCCCCCCCchHHHHHHHHHHhCCCh----hHHHHHHHHHHHH----HHHHhhhc----

Q ss_pred         CccccCCCccchhhhhhccccCCCCCCCCCccchHHHHHHHHHHHHHHH----HHHHHHHHHHHhccCCCCccccCCCCcC
Q 1           116 SVTFLVGIPKIGSFLNRIAFGVVDSGRRPFPLKNVFPIMTIRFLTSYAV----VMVFRTFVIMGTSYPATDNHCQNPQVI   191 (385)
Q Consensus   116 ~~~~~~~~~~p~v~~~~~~~~w~~~~~~~~~~~~~~~~~~~~~~~ii~rR~l~~~~i----lyllR~iti~vT~LP~P~~~C~~~~~~~~   191 (385)
                  .....+++....+ ..++-.+....+..|.|.....+....
T Consensus    72 ------------------------------------~~~~~~~~~~~~~~~~~~~~~~~~~~lk~~~~~~rP~~~~~~~~~~~~   115 (259)
T 5JWY_A      72 ------------------------------------IKAAFVLFAILAAAILVGQGVKSWIKDKVQEPRPFVIWLEKTHH   115 (259)
T ss_dssp        ------------------------------------HHHHHHHHHHHHHHHHHHHHHHHHHHHHTTTCCCCCHHHHHHHHHTT
T ss_pred        ------------------------------------HHHHHHHHHHHHHHHHHHHHHHHHHHHHHHhCCCCCeeHHHcccCC

Q ss_pred         CCcHHHHHHHH-------------------HHhhcCCCccccchhHHHHHHHHHHHHHHHHHHcCCcccchHHHHHHHHHHHH
Q 1           192 EHPVLNVILT-------------------LVTLSSGAIHCGDLMFSGHTMILSLAFILAWDYSPFLHPWAVRVWVSALLP   252 (385)
Q Consensus   192 ~~~~~~~~~i~~-------------------~l~~g~~~~~CgDlifSGHT~~~~l~~l~~~~y~~~~~~~~~~~~~~~i~~~~~~~~   252 (385)
                  .......... ....+.....-+..++|||+........++..+..+..++....+..+..+
T Consensus   116 ~~~~~~~~~~~~~~~~~~~~~~~~~~~~~~~~~~~~~~~~~~~fPSgh~~~~~~~~~~~~~~~~~~~~~~~~~~~~~~~~~~~   195 (259)
T 5JWY_A     116 IPVDEFYTLKRAERGNLVKEQLAEEKNIPQYLRSHWQKETGFAFPSGHTMFAASWALLAVGLLWPRRRTLTIAILLVWAT   195 (259)
T ss_dssp        CCHHHHHSSCHHHHHHHHHHHHHHHHCCSSCHHHHHHHHHHHTTSCCCCHHHHHHHHHHHHHCCCCCGGGTCHHHHHHHHHHHH
T ss_pred        CChHHHhhccHHHHHHHHHHHHHHHccCCCCHHHHHhhhccCCCCCCHHHHHHHHHHHHHHHHHHHHCcccHHHHHHHHHHHHHH

Q ss_pred         HHHHHHHHhcccccHHHHHHHHHHHHHHHHHHHHHccCCchhhHHHHHhccCCCCCcc
Q 1           253 ISYYCILASRSHYTDDILVAMYVMIATYKLIDHAETGAPWQMQLLIRWMPWPGTNAI   309 (385)
Q Consensus   253 ~~~~~~ii~~R~HYTIDVi~a~~it~~~~~~~~y~~~~~~~~~~~~~~~~~~~l~~ww~~~~~~~~~~~   309 (385)
                  ...+..++.+.||-.|++.|..++.....+. ....+++.+......
T Consensus   196 ~~~~~s~v~~~~~H~~~Dv~~g~~lg~~~~~~~~~-----------~~~~~~~~~~~~~~~~~~~   241 (259)
T 5JWY_A     196 GVMGSRLLLGMHWPRDLVVATLISWALVAVA-----------TWLAQRICGPLTPPA   241 (259)
T ss_dssp        HHHHHHHHHTCCCHHHHHHHHHHHHHHHHHHHH-----------HHHHHHHHCCCSCCH
T ss_pred        HHHHHHHHhCCCcHHHHHHHHHHHHHHHHHHHH-----------HHHHHHHhCCCCCCh
```

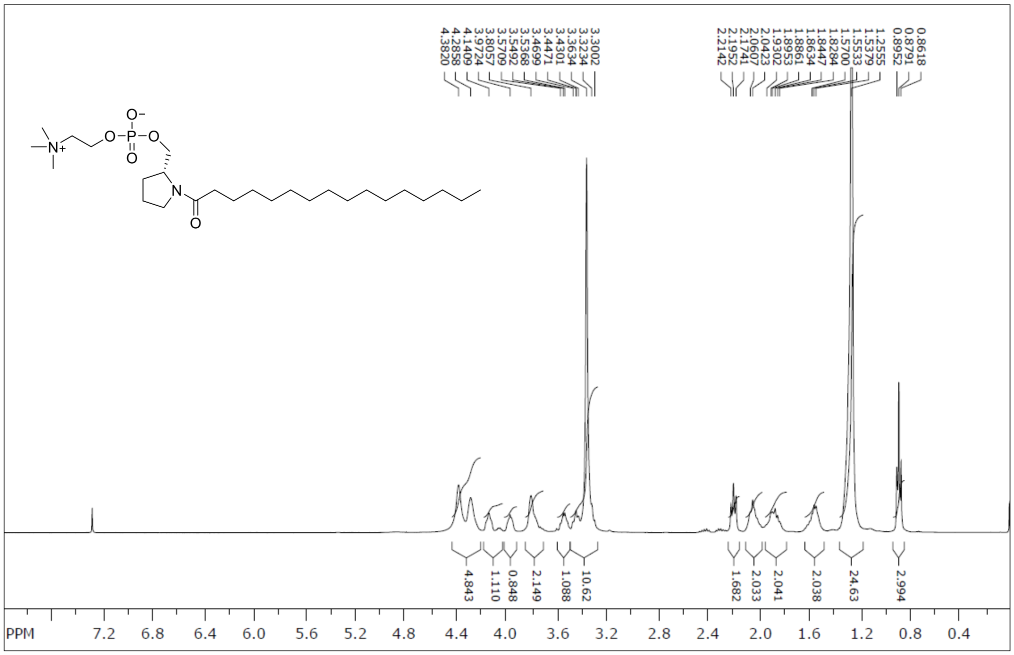

^{1}H NMR spectra of compound **1a**

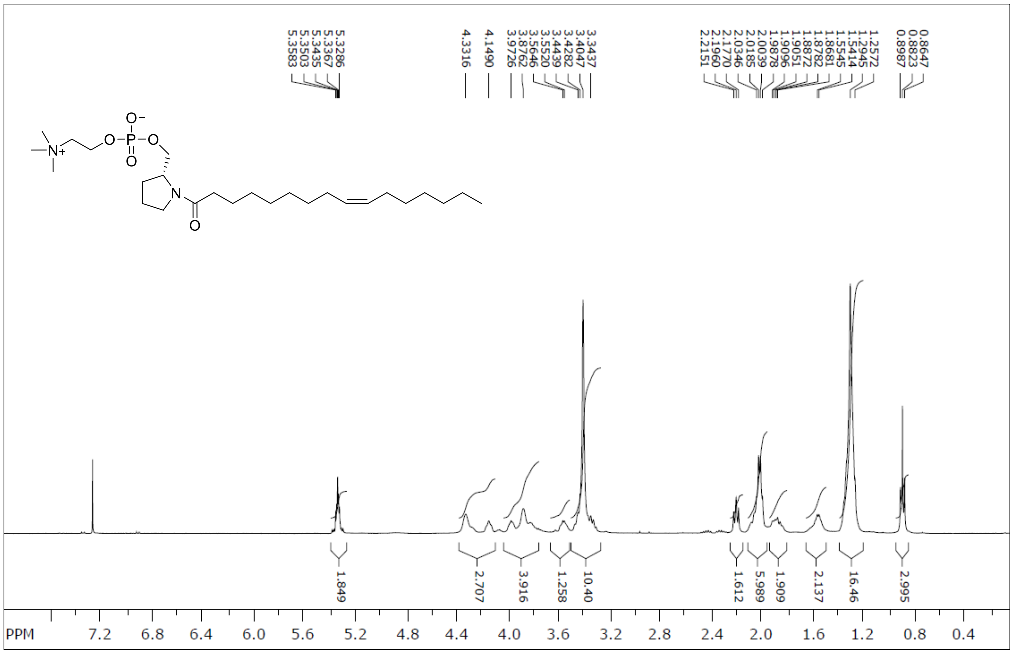

^{1}H NMR spectra of compound **1b**

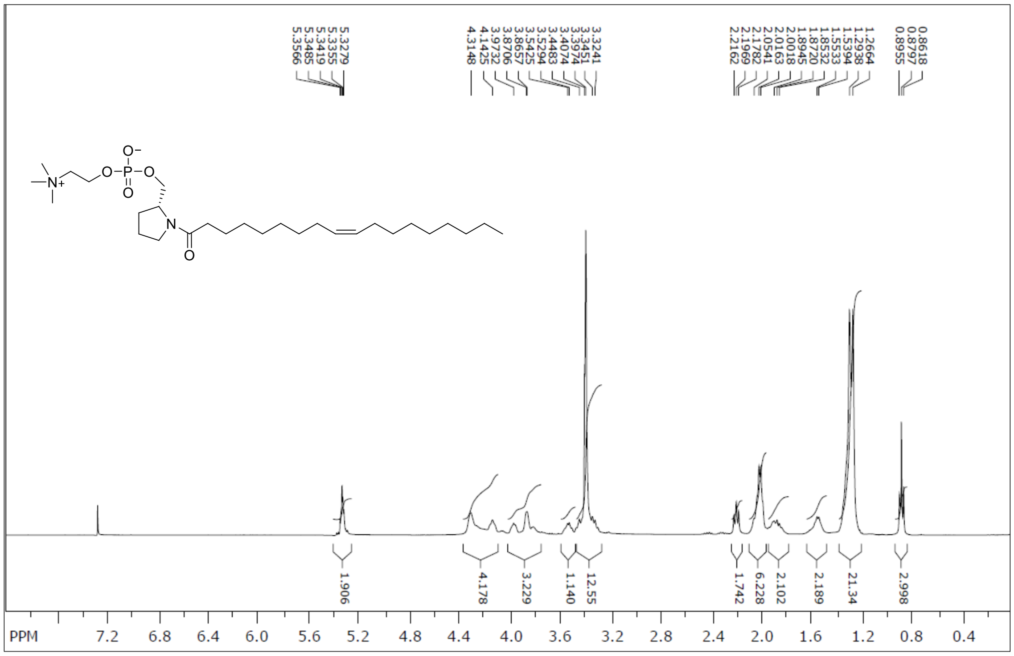

^{1}H NMR spectra of compound **1c**

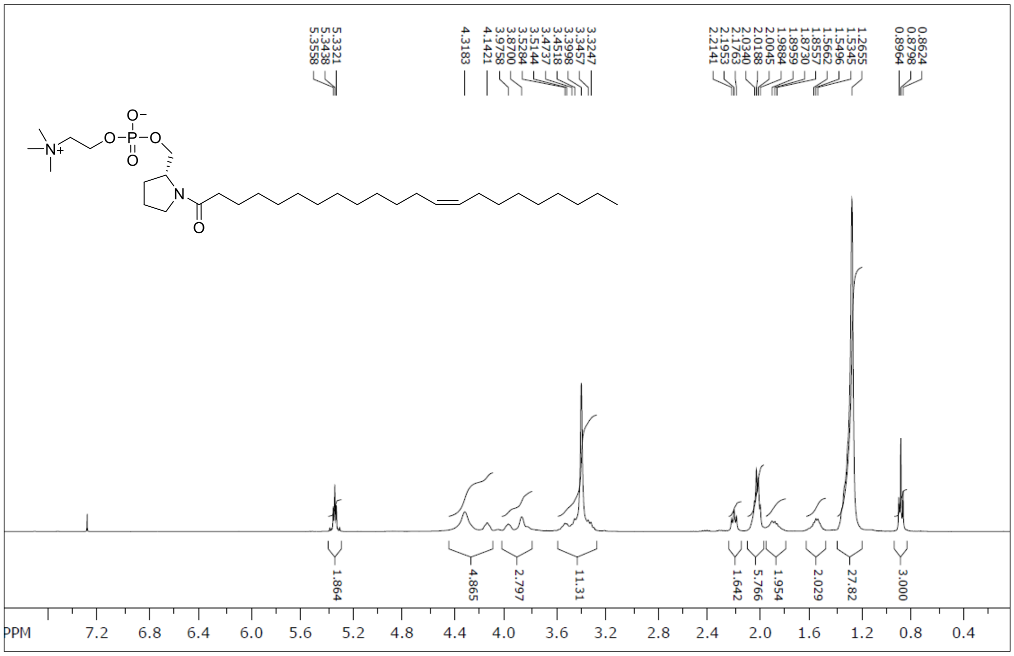

^{1}H NMR spectra of compound **1d**

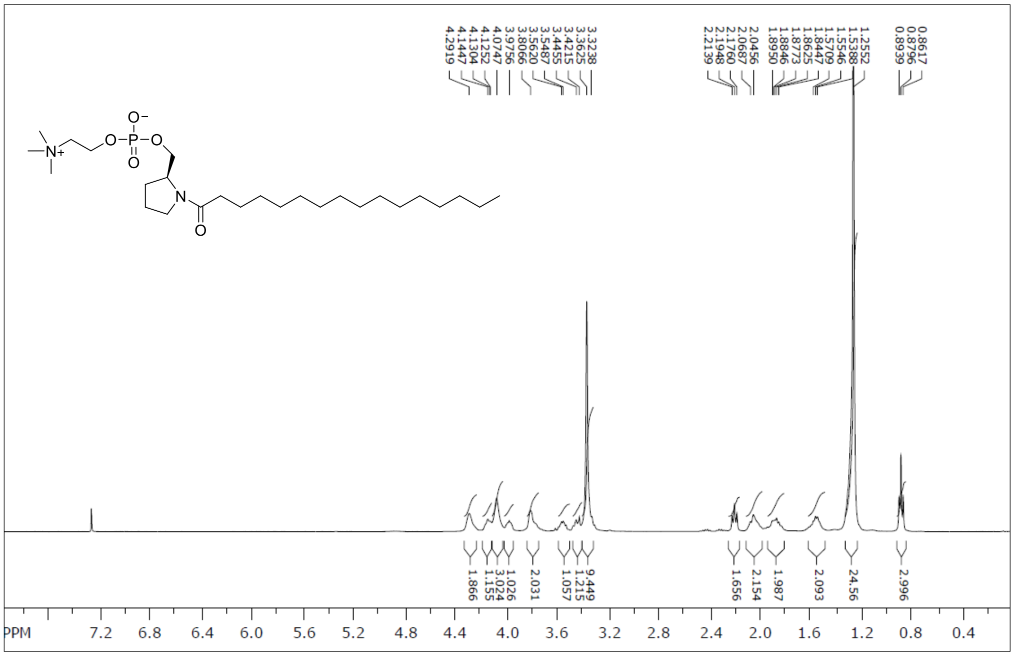

^{1}H NMR spectra of compound **1e**

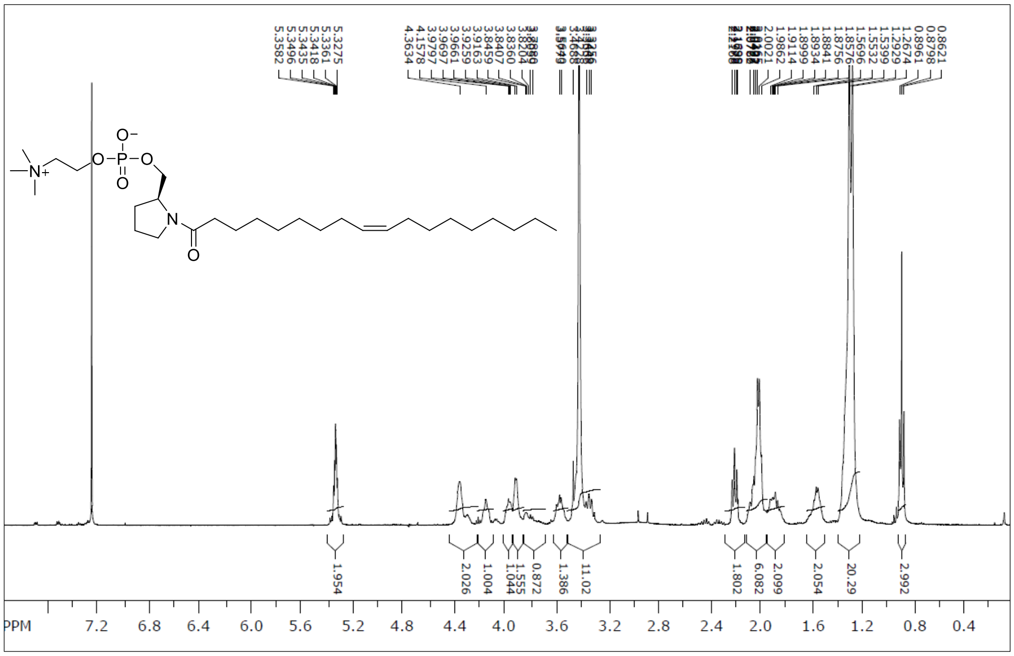

^{1}H NMR spectra of compound **1f**

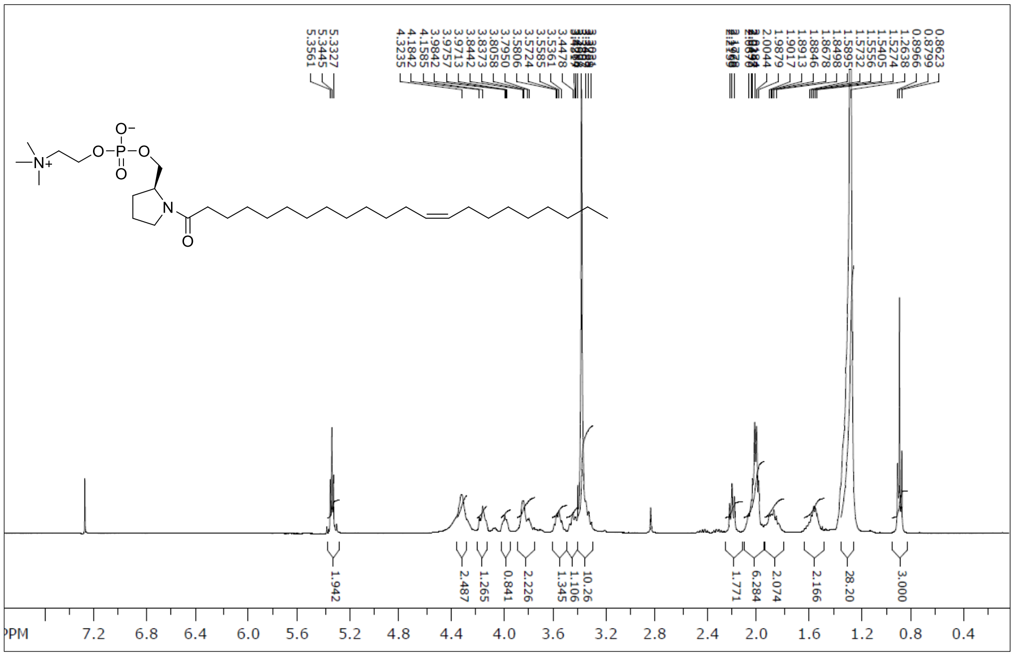

^{1}H NMR spectra of compound **1g**

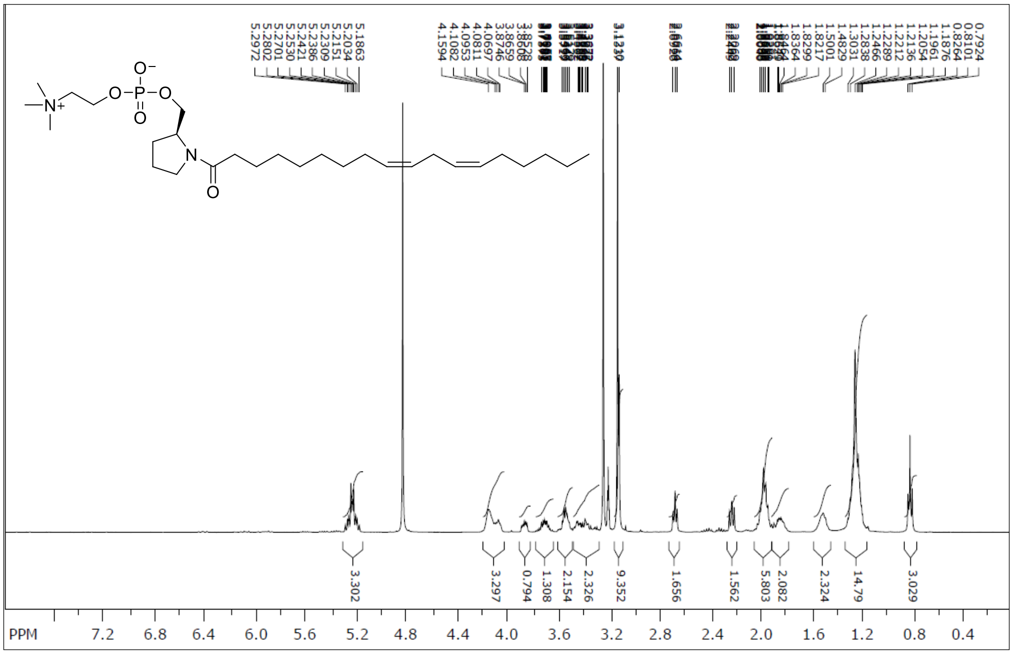

^{1}H NMR spectra of compound **1h**

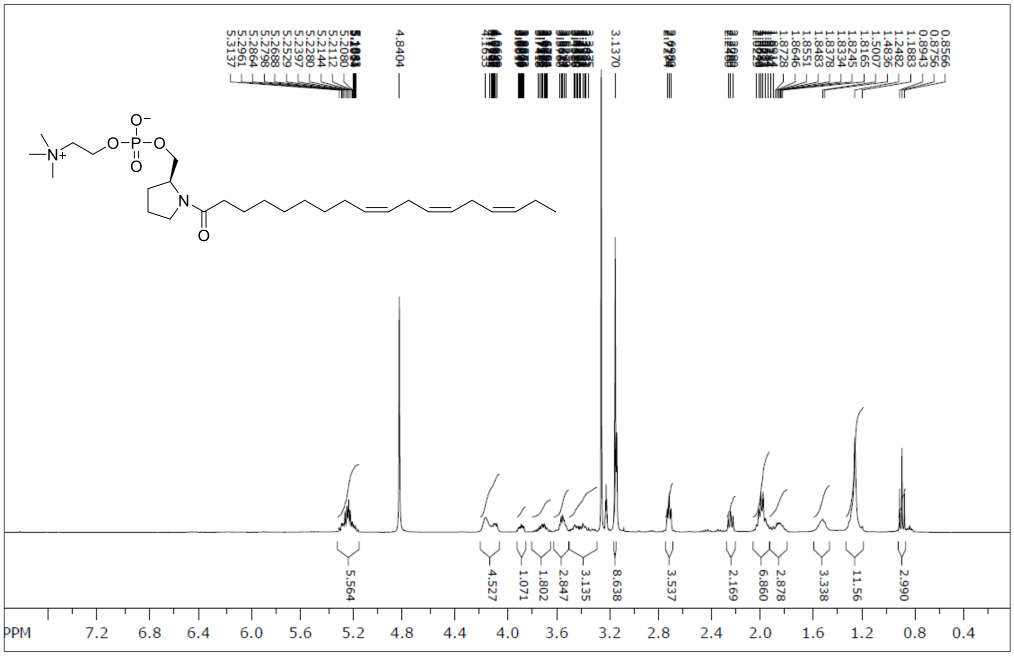

^{1}H NMR spectra of compound **1i**

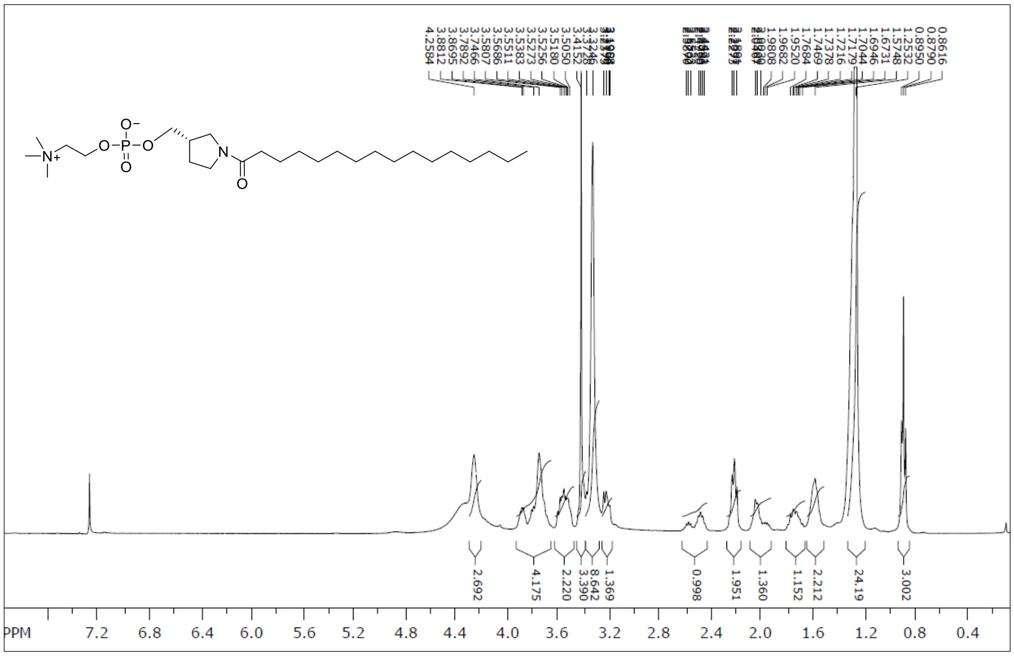

^{1}H NMR spectra of compound **2a**

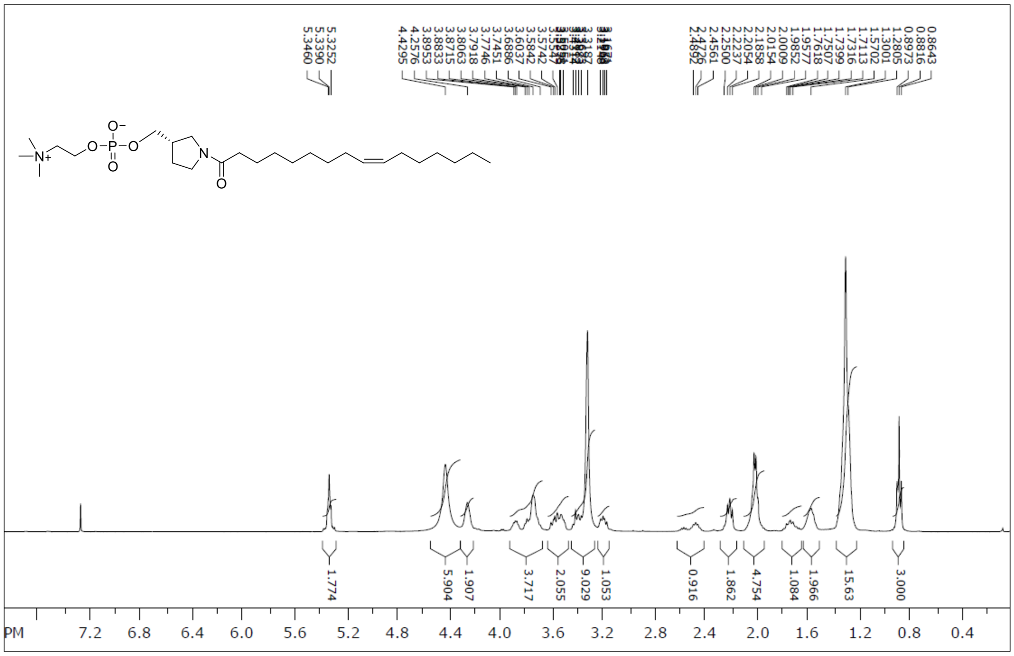

^{1}H NMR spectra of compound **2b**

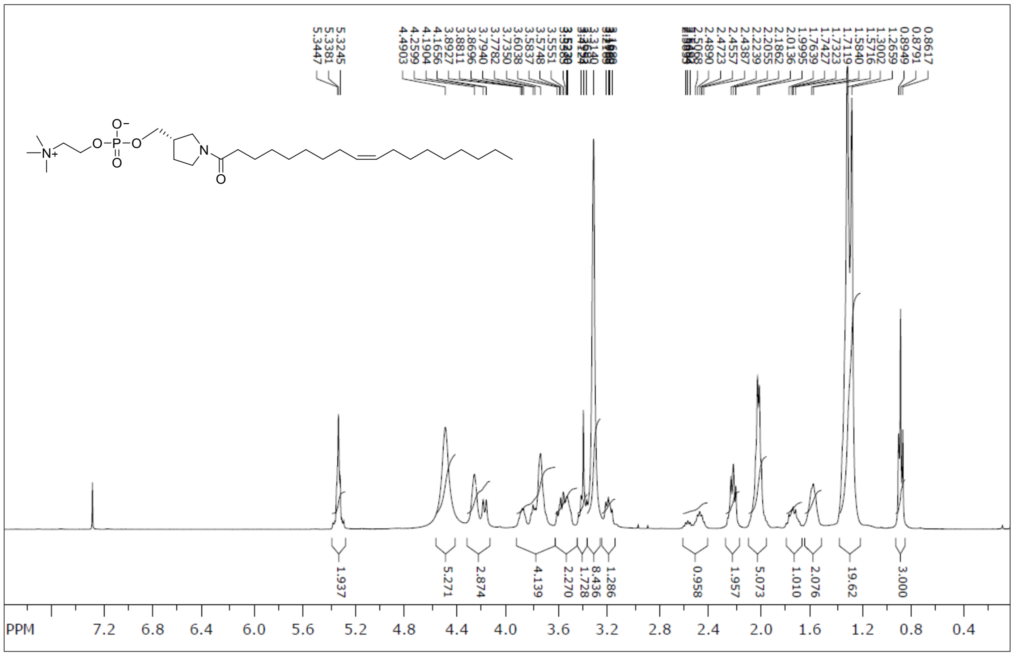

^{1}H NMR spectra of compound **2c**

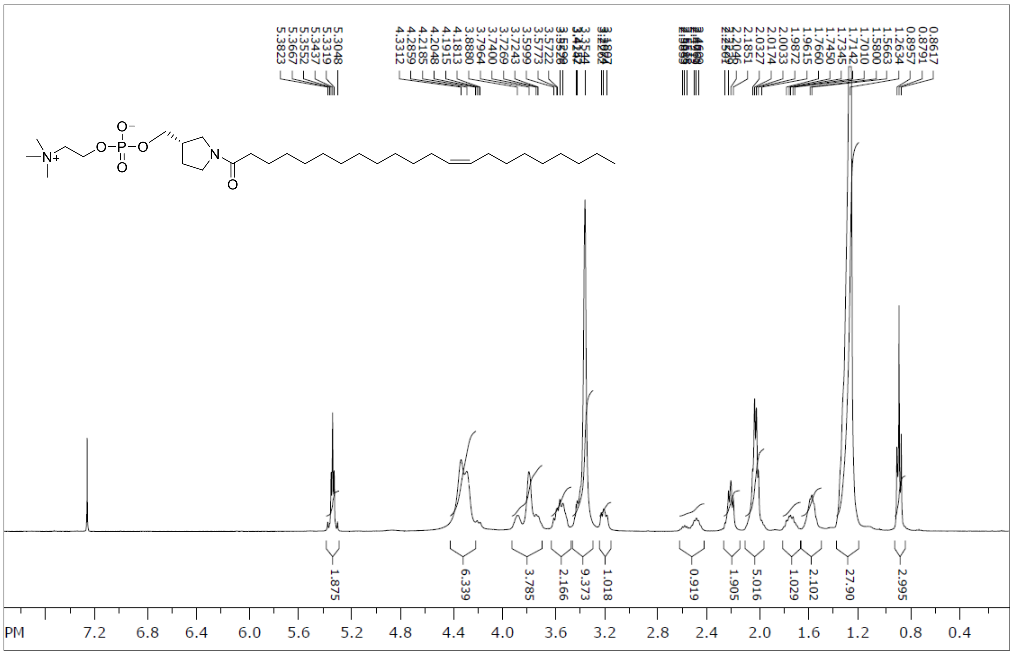

^{1}H NMR spectra of compound **2d**

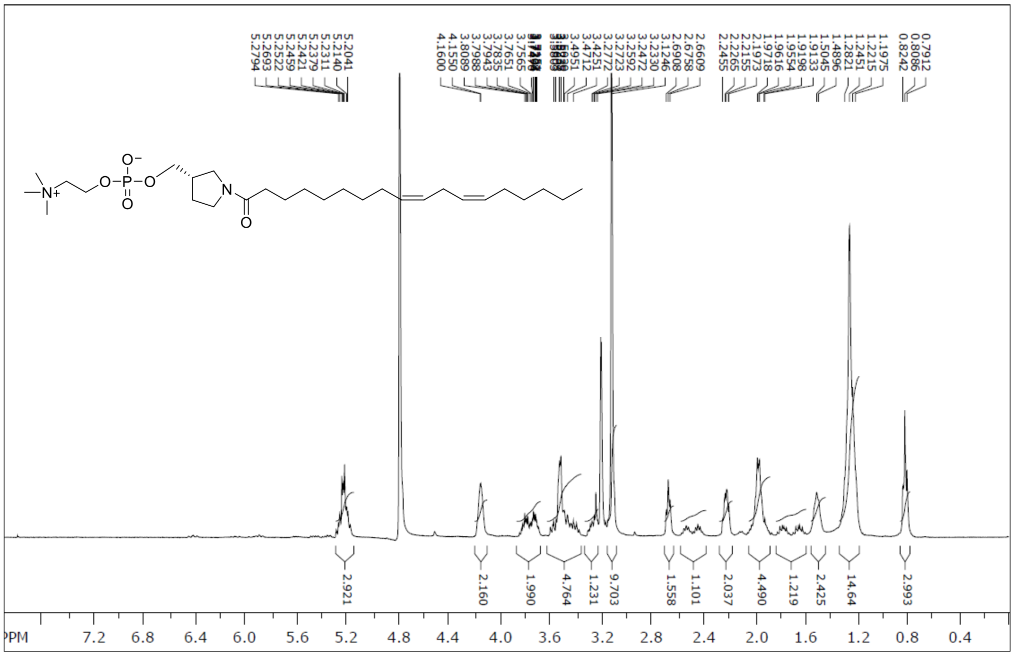

^{1}H NMR spectra of compound **2e**

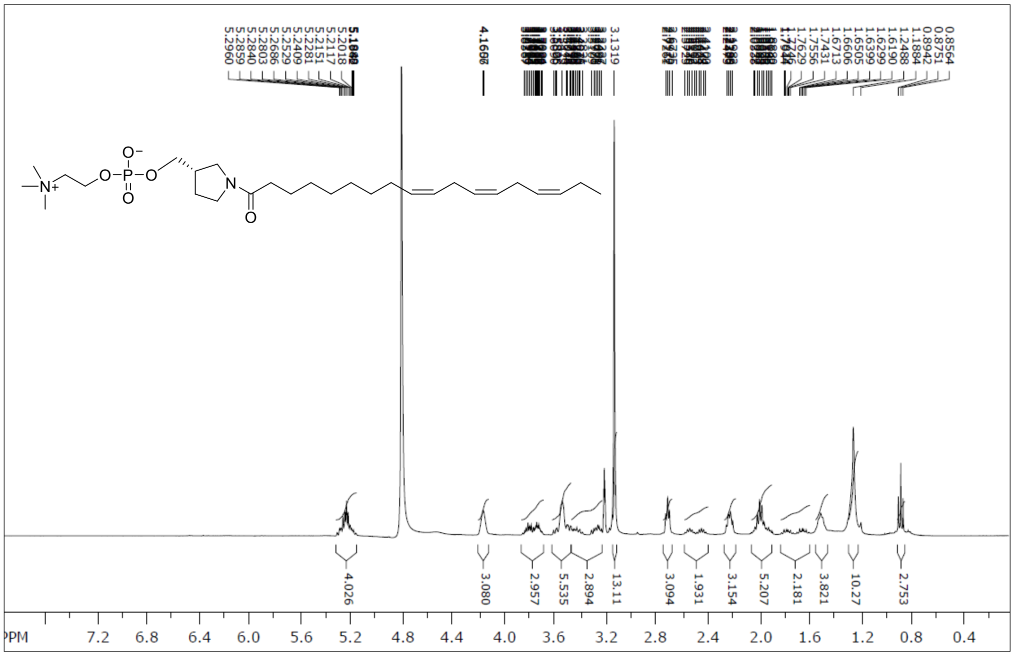

^{1}H NMR spectra of compound **2f**

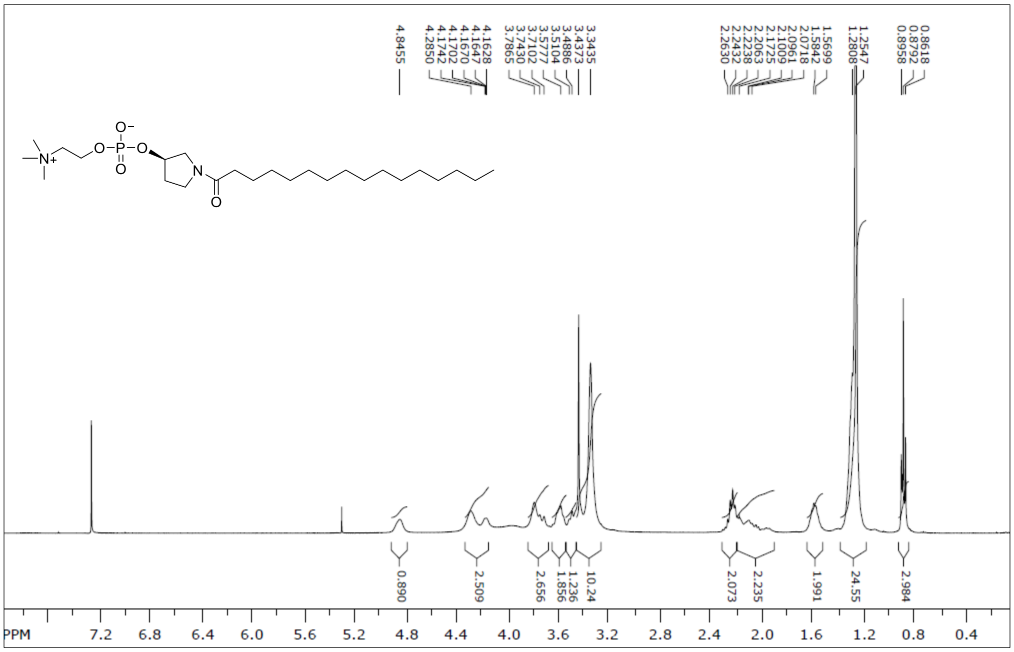

^{1}H NMR spectra of compound **3a**

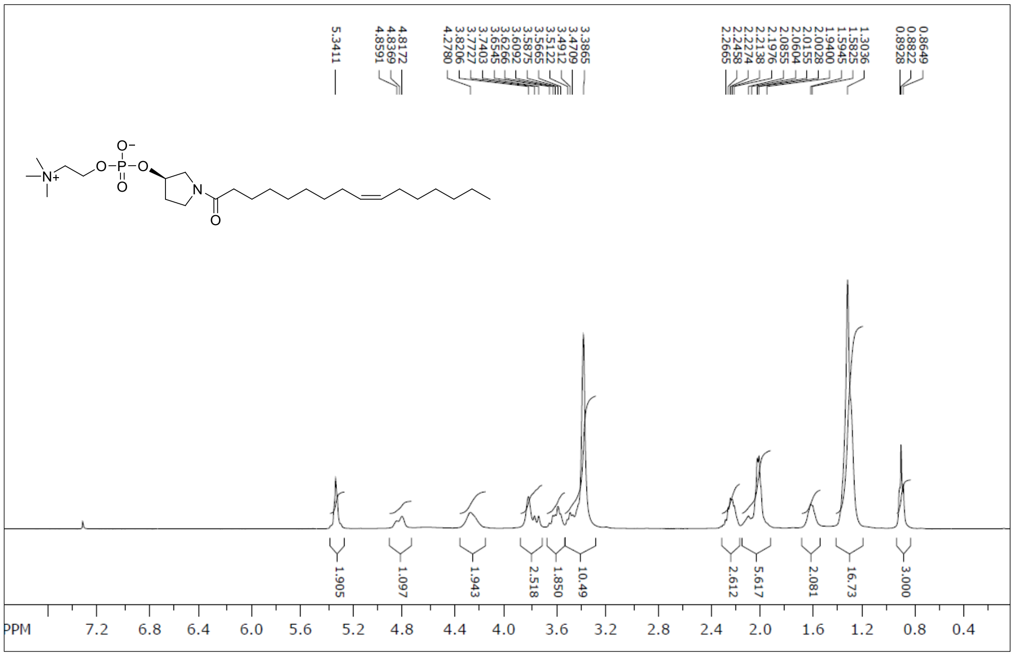

^{1}H NMR spectra of compound **3b**

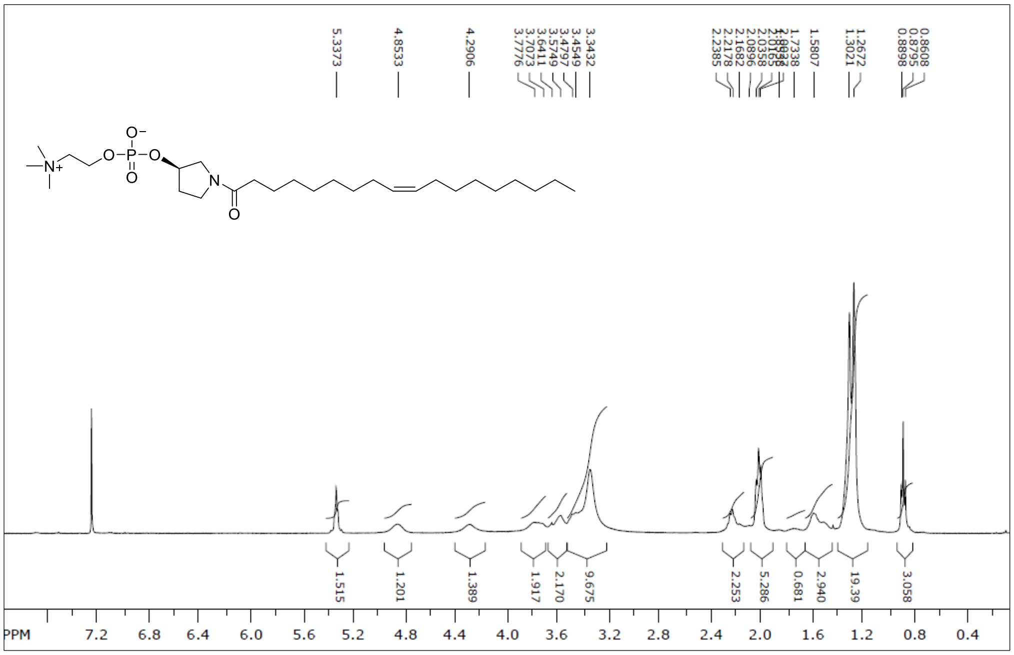

^{1}H NMR spectra of compound **3c**

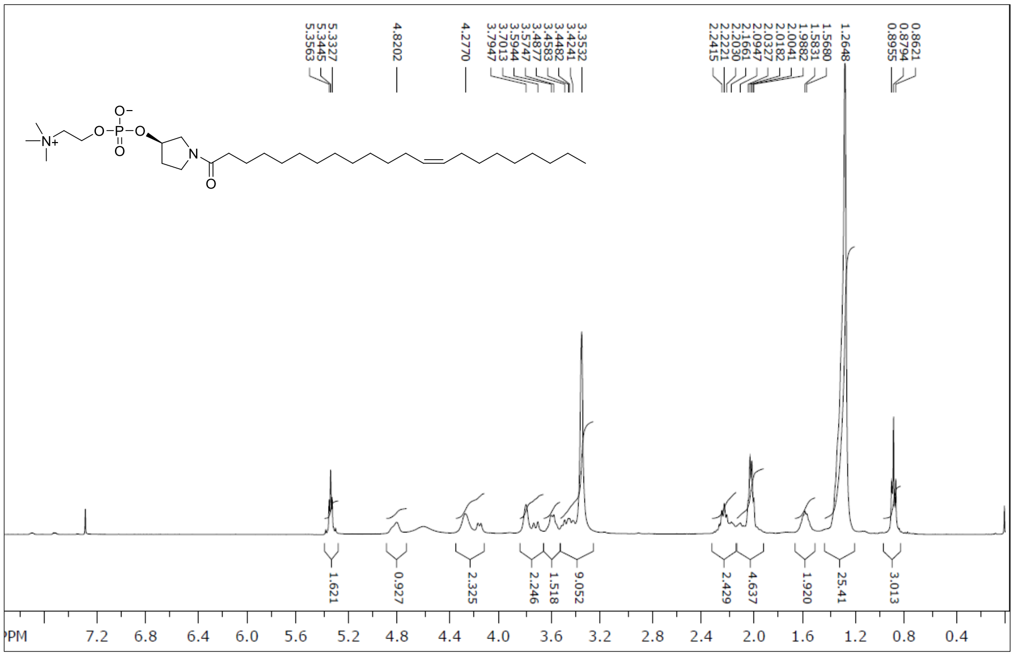

^{1}H NMR spectra of compound **3d**

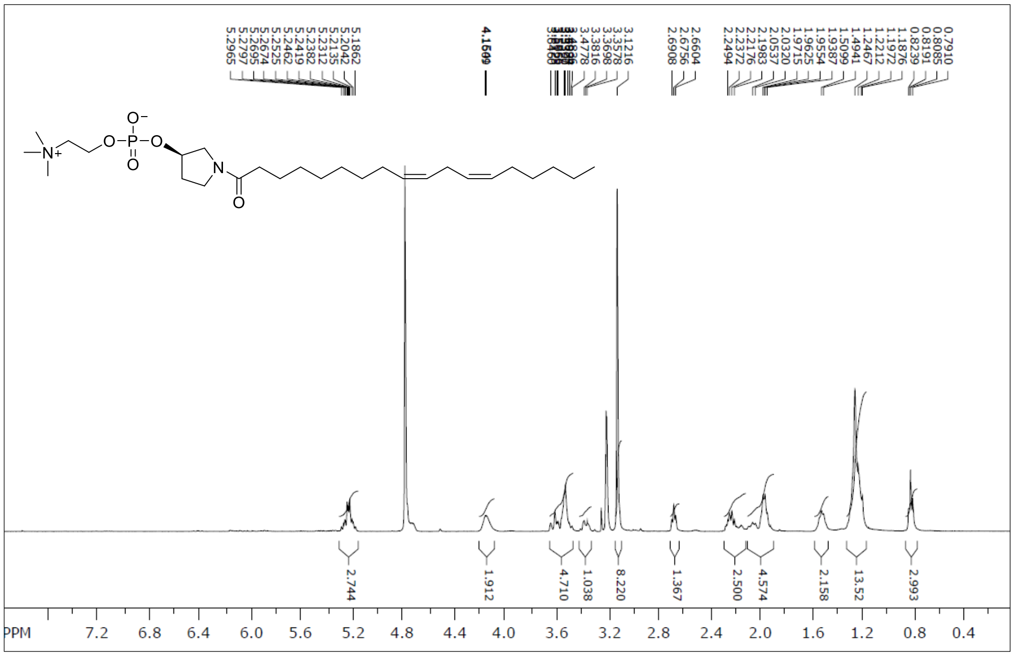

^{1}H NMR spectra of compound **3e**

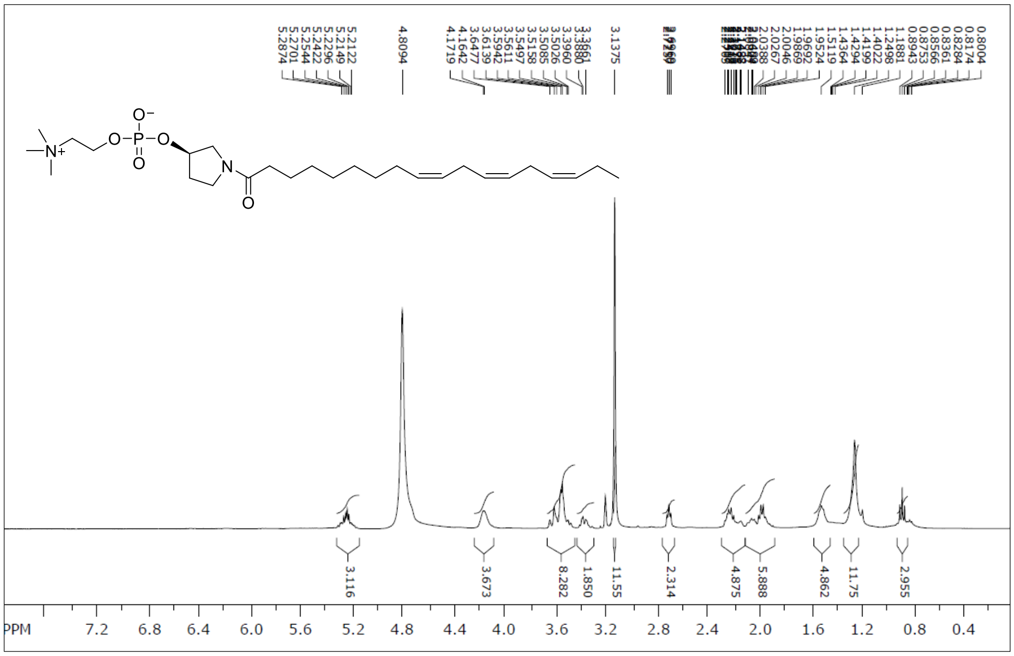

^{1}H NMR spectra of compound **3f**
